# Structure and organization of AMPA receptor-TARP complexes in the mammalian cerebellum

**DOI:** 10.1126/science.aeb3577

**Published:** 2025-12-11

**Authors:** Alexander M. Scrutton, Nayanika Sengupta, Josip Ivica, Imogen Stockwell, Sew Peak-Chew, Bishal Singh, Kunimichi Suzuki, Veronica T. Chang, Stephen H. McLaughlin, James M. Krieger, A. Radu Aricescu, Ingo H. Greger

**Affiliations:** 1 Neurobiology Division, Medical Research Council (MRC) Laboratory of Molecular Biology, Cambridge CB2 0QH, UK; 2 Cell Biology Division, Medical Research Council (MRC) Laboratory of Molecular Biology, Cambridge CB2 0QH, UK; 3 Department of Genomic Medicine; University of Texas MD Anderson Cancer Center Houston 77030, Texas, USA; 4 Department of Physiology, Keio University School of Medicine, Tokyo 160-8582, Japan; 5 PNAC Division, Medical Research Council (MRC) Laboratory of Molecular Biology, Cambridge CB2 0QH, UK

## Abstract

AMPA receptors (AMPARs) are multimodal transducers of glutamatergic signals throughout the brain. Their diversity is exemplified in the cerebellum; at afferent synapses, AMPARs mediate high-frequency excitation, whereas in Bergmann glia (BG) they support calcium transients that modulate synaptic transmission. This spectrum arises from different combinations of core subunits (GluA1-4), auxiliary proteins, and post-transcriptional modifications. Here, using mass-spectrometry, cryo-EM, and electrophysiology, we characterize major cerebellar AMPARs in pig: calcium-impermeable GluA2/A4 heteromers with four TARP subunits, mainly neuronal in origin, and BG-specific calcium-permeable GluA1/A4 heteromers containing two Type-2 TARPs. We also showed that GluA4 receptors frequently exhibit compact N-terminal domains that promote their synaptic delivery. Our study defines the organizational principles of mammalian cerebellar AMPAR complexes and reveals how different receptor subtypes support cell-type specific functions.

## Introduction

AMPARs respond to glutamate to enable fast excitatory synaptic transmission throughout the nervous system (1). Their molecular diversity facilitates circuitry-specific synaptic signaling and plasticity mechanisms, fundamental to learning and memory (1–5). The four AMPAR core subunits (GluA1-4), modifiable by alternative splicing and RNA editing, assemble into homo- or hetero-tetramers that vary in gating kinetics and calcium permeability (6). These are diversified further by an array of auxiliary subunits. TARPs (transmembrane AMPAR regulatory proteins), which are differentially expressed across brain regions, form the main group of auxiliary subunits shaping many aspects of AMPAR function (1, 6, 7).

AMPARs in the forebrain are mostly GluA2-containing, and thus of low calcium-permeability (‘calcium-impermeable’; CI) (1, 8). Typically, two GluA2 subunits occupy the inner B/D positions of the receptor tetramer (9, 10), with GluA1 and/or GluA3 at the outer A/C sites. Additionally, two TARP and two CNIH (cornichon homolog) auxiliary subunits occupy the four available binding sites (11, 12). The non-stochastic B/D placement of GluA2 stabilizes the receptor through an inter-dimer interface between the GluA2 N-terminal domains (NTDs), facilitating synaptic receptor delivery and synaptic transmission (13–15).

**Fig. 1:**
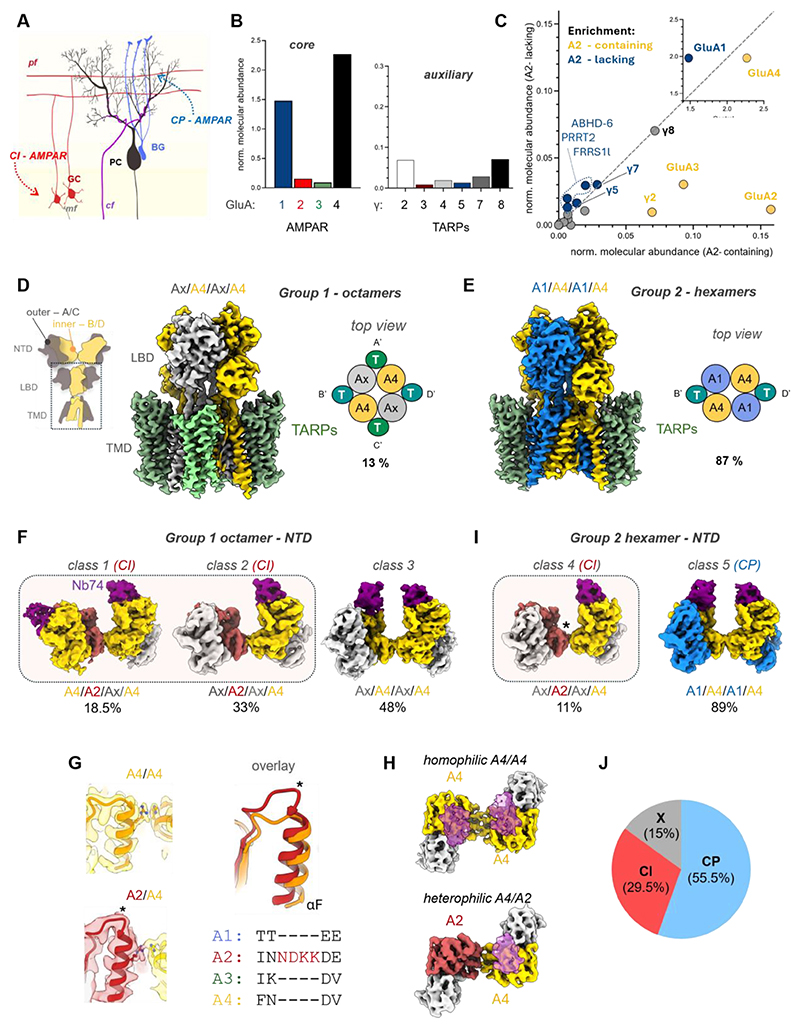
Diversity of cerebellar CP and CI GluA4-AMPARs. (**A**) Simplified schematic of the cerebellar cortex circuitry. The major mossy fiber (MF) to granule cell (GC) input operates via CI AMPARs, while Purkinje cells (PCs) are modulated by Bergmann glia (BG), requiring CP AMPAR-dependent calcium transients. (**B**) Semi-quantitative MS from GluA4 pulldowns from pig synaptosomes. Core (A1-A4) and auxiliary (TARPs) subunits are shown as a ratio of total AMPAR core-subunit abundance. (**C**) Enrichment of core and auxiliary subunits in A2-depleted samples (y-axis) compared to A2-containing (x-axis). (**D**) Inset: AMPAR schematic, outlining main chain nomenclature. LBD-TMD tier of group 1 octamers, with 4 auxiliary TARP subunits. A4 generally occupies the B’D’ positions of the tetramer, while A’C’ positions are unassigned. (**E**) LBD-TMD layer of group 2 hexamers, with 2 TARPs. A4 generally occupies the B’D’ positions, while A’C’ positions are occupied by A1. (**F**) Major classes of group 1 based on NTD-focused classification. (**G**) Left: Homophilic A4/A4 and heterophilic A2/A4 NTD tetramer interfaces. Right: The A2 subunit exhibits unique residues at the extended loop between helix F and β-strand 7, outlined in the sequence alignment. (**H**) Top view, comparing the difference in the A4/A4 (homophilic) and A4/A2 (heterophilic) tetrameric NTD interface. (**I**) Major classes of group 2 based on NTD-focused classification. (**J**) Overall distribution of A4-containing CP and CI AMPARs in cerebellum based on cryo-EM particle numbers.

Although sparsely expressed in the forebrain, GluA4 is selectively enriched in the cerebellum (16–18). Here, GluA4 exists in both CI and calcium-permeable (CP) AMPARs, segregating into neurons and glia with distinct functions. A major cerebellar afferent pathway, formed between mossy fibers and granule cells (GCs) (Fig. 1A), is mediated by CI GluA4 AMPARs (19). GluA4 deletion blunts transmission at this synapse and leads to deficits in associative learning (20). The sole output of the cerebellar cortex is formed by Purkinje cells (PCs), where synaptic activity is tightly regulated by Bergmann glia (BG). BG rely exclusively on CP AMPARs, whose signals orchestrate diverse functions through glia-neuron coupling (21–24) (Fig. 1A). TARP auxiliary subunits similarly exhibit cell type-specific functions across cerebellar circuits (25, 26). Deletion of TARP-γ2 (stargazin) abolishes GC-mediated transmission, resulting in ataxia and epilepsy in the stargazer mouse (27, 28). Thus, the cerebellar network employs diverse GluA4 and TARP combinations to orchestrate its multiple synaptic computations.

Using cryo-EM, mass-spectrometry (MS) and electrophysiology, we unraveled the organization of predominant GluA4-TARP AMPAR complexes, both CP and CI subtypes, isolated from pig cerebellum. Moreover, we demonstrate further segregation in the properties of these receptor complexes by both TARP subtype and stoichiometry.

## Results

### The GluA4 cerebellar proteome

The selective enrichment of GluA4 in the cerebellum is well-established (16, 18), and single-cell RNAseq data confirms its wide distribution across distinct cerebellar cell types (fig. S1A) (17). As the pig proteome matches the human proteome more closely than that of mouse (29), we sought to investigate the molecular composition and structure of AMPAR complexes from pig cerebellar synaptosomes (Methods) (fig. S1B). To characterize GluA4-containing complexes, we isolated a nanobody (Nb74) that selectively recognizes the GluA4 extracellular N-terminal domain (NTD), the most sequence-diverse portion of AMPAR subunits, from a library raised against GluA receptor subunits. Nb74 interacts with the upper lobe of the NTD with pico-molar affinity and is highly selective for GluA4 (fig. S1C-D).

We solubilized AMPARs with either lauryl maltose neopentyl glycol (LMNG) or with digitonin, which preserves AMPAR-TARP complexes (30). When subjected to mass spectrometry (LC-MS-MS), GluA4 was heavily enriched in both samples (relative to the sum of GluA1-A4), closely followed by GluA1. GluA2 and GluA3 co-purified with GluA4 to a far lesser extent (Fig. 1B), mirroring the proteome analysis from rat cerebellum (18). The GluA4 receptor core is dominated by TARPs, while peptides for other auxiliary subunits were sparser (Fig. 1B and fig. S1E-H). Contrasting with forebrain AMPARs (18), cornichon subunits were mostly undetected (fig. S1H). Among the six TARP subunits (γ2, γ3, γ4, γ5, γ7, γ8) (31), the Type-I TARPs γ2 and γ8 prevailed together with γ7 (a Type-II TARP) (32). Although γ8 predominantly concentrates in the cortex and hippocampus in rodent brain (31), this TARP has been shown to associate with cerebellar GluA4 (18).

We observed a relationship between core subunit and TARP subtype: depletion of GluA2 (prior to GluA4 purification) co-depleted TARPs-γ2 and -γ3, leaving the remaining TARPs and GluA1 mostly unaffected (Fig. 1C and fig. S1F). Moreover, extraction with LMNG strongly enriched GluA2 together with TARP-γ2 (fig. S1G), likely through improved extraction from synaptic scaffolds. This apparent relationship between core subunit and TARP subtype is in line with observations from TARP knock-out mice, where deletion of γ2 specifically reduces GluA2 amounts (26, 33). Furthermore, co-immunoprecipitation data showed a preferential association between GluA1/A4 with TARP-γ7 (fig. S1I). The co-existence of GluA2 and GluA3 AMPARs with TARPs γ2 and γ3 likely relates to specific expression patterns of AMPAR proteome components across cerebellar cell-types (17): GluA2, GluA4 and TARP-γ2 are mostly co-expressed in cerebellar interneurons and in the highly abundant GCs.

### Two major cerebellar AMPAR-TARP complexes

To elucidate the organization of cerebellar AMPARs, we subjected Nb74-isolated GluA4, solubilized in digitonin and trapped in a resting state with the antagonist NBQX, to structural analysis by cryo-EM (sample preparation matched that of MS). Initial 3D classification yielded two groups of receptors, associated with either four (group 1), or two (group 2) auxiliary subunits resembling TARPs (Fig. 1D, E and fig. S2A), consistent with our MS data (fig. S1E-H). The ‘fully TARPed’ octameric group 1 was less abundant than the two-TARP hexamers of group 2 (13 % vs 87%). Together with proteomic (18), and functional data (34), this suggests that a large proportion of cerebellar AMPARs lack auxiliary subunits at their A’/C’ binding sites (Fig. 1E). Auxiliary subunit stoichiometry is a crucial determinant for AMPAR operation, as four TARPs enable greater charge transfer though the channel (6, 7), and facilitate anchoring to the PSD-scaffold (14, 35). All four sites are saturated in hippocampal, cortical and various recombinant AMPARs under comparable purification conditions (10–12, 36–38). Annular lipids occupy the TARP-free A’/C’ binding-sites in group 2 (see below), further demarcating the structural difference between the two main receptor types.

### AMPAR octamers contain GluA2

We next tested whether Nb74 reliably reports GluA4 subunit stoichiometry. To do this, we isolated recombinant GluA4-flip, co-expressed with TARP-γ2 in HEK293-Expi cells, with Nb74 and subjected the sample to analysis by cryo-EM (Methods). As shown in fig. S3A, the 3D class exhibiting the best-resolved NTD tier featured four Nb74 densities emanating from the NTD upper lobes of the receptor tetramer. Nb74 signal intensity was comparable across the four subunits (fig. S3B), and together with its high specificity (fig. S1D), was suitable to report subunit stoichiometry of native GluA4 receptors.

Nb74 facilitated further classification of core subunits in groups 1 and 2 (fig. S2B). We first characterized group 1 octamers, where a focused classification of the Nb74-labelled NTD tier yielded four classes with either a single ‘inner’ (B or D) GluA4 subunit (see Fig. 1D, inset), or with two GluA4 molecules segregated between the two NTD dimers in various constellations (fig. S4A). To address compositional heterogeneity further, we conducted symmetry expansion of the NTD tier, followed by focused 3D classification. This procedure generated three major types of classes (Fig. 1F, fig. S4A): class 1, where two GluA4 subunits locate at one inner and one outer site (~ 19%), class 2, containing a single inner GluA4 subunit (~ 33%), and class 3, exhibiting two GluA4 subunits at both inner sites (~ 48%) (fig. S4A). Of note, the stoichiometry and position of GluA4 will determine the arrangement of the agonist coordinating LBDs (1) - class 1 receptors contain GluA4 LBD homodimers (fig. S4A), thus conferring a different kinetic profile to receptors in the two other classes.

In all three classes, an ordered, tetrameric NTD tier was formed by an interface between the B/D subunits, composed of either A4/A4 or A4/Ax (Fig. 1F and fig. S4A). To identify the Ax subunit we refined classes 1 and 2 to ~4 Å, revealing GluA2 as the other inner subunit, based on its uniquely extended loop between helix F and β-strand 7 (Fig. 1G and fig. S4B). Therefore, approximately 50% of group 1 receptors are GluA2-containing, thus of low Ca^2+^-permeability (1, 8), and with a preferred arrangement: the B/D positions are occupied by GluA2 and GluA4, engaging a heterophilic NTD interface (Fig. 1F, H).

Occupancy of the four TARPs is not equal across group 1. The A’/C’ TARPs, located beneath the LBD dimers, exhibit weaker signal than the B’/D’ TARPs, positioned *between* the LBD dimers (fig. S4C). This likely results from spatial restriction imposed by the LBD dimers onto A’/C’ TARPs, as observed earlier in recombinant AMPARs (12, 39). To better resolve the TARP sector we conducted a further focused classification and refinement leading to improved B’/D’ TARPs, which could be fitted satisfactorily with Type-I TARP models (fig. S4D). Based on the MS data (Fig. 1C and fig. S1E-H) and on RNAseq (17), we propose that the GluA2/A4 octamers are largely associated with TARP-γ2, and these most likely derive from the abundant GCs known to signal through CI AMPARs (20, 25, 27).

### Hexamers are CP GluA1/4 receptors

The same processing procedure revealed that group 2 hexamers (Fig. 1E, fig. S5A) predominantly harbor two inner GluA4 subunits and two outer GluA1 subunits, marked by two specific N-glycans at Asn45 and Asn239 (class 5, ~ 89 %) (fig. S5B). Hence, group 2 receptors are largely CP GluA1/4 heteromers at a 2:2 stoichiometry, associated with two TARPs at the B’/D’ sites. The remaining ~11 % of group 2 (class 4) contain a single inner GluA4 subunit interfacing with GluA2 (Fig. 1F-H and fig. S5C), further highlighting the prevalence of this GluA2/A4 subunit arrangement in cerebellar AMPARs (Fig. 1J).

### The GluA1/4 BG receptor harbors Type-II TARPs

GluA1 expression in the cerebellum is predominantly confined to BG (17, 21, 40), forming a receptor whose calcium signal is essential for PC synapse function and anatomy (22–24). Class 5 may therefore represent this specialized CP-AMPAR. To better resolve this receptor, and to further clarify the relationship between CI AMPARs and TARP stoichiometry, we depleted GluA2 from the cerebellar extract, added the LY-481 ligand to selectively mark TARP-γ8 complexes (41, 42), and purified GluA4 with Nb74 (fig. S6A). Two independent cryo-EM datasets were indeed dominated by hexamers with two inner GluA4 subunits (fig. S6B). This finding further supports the association of GluA2-containing receptors with four TARPs, as these receptors were effectively removed from this preparation (Fig. 1C, fig. S1F). To facilitate further subunit characterization, we combined particles featuring two B/D GluA4 subunits and GluA1 N-glycan signal at the A/C sites and performed refinements of the individual receptor tiers (fig. S6B, C).

#### The GluA1/4 NTD tier

**Fig. 2:**
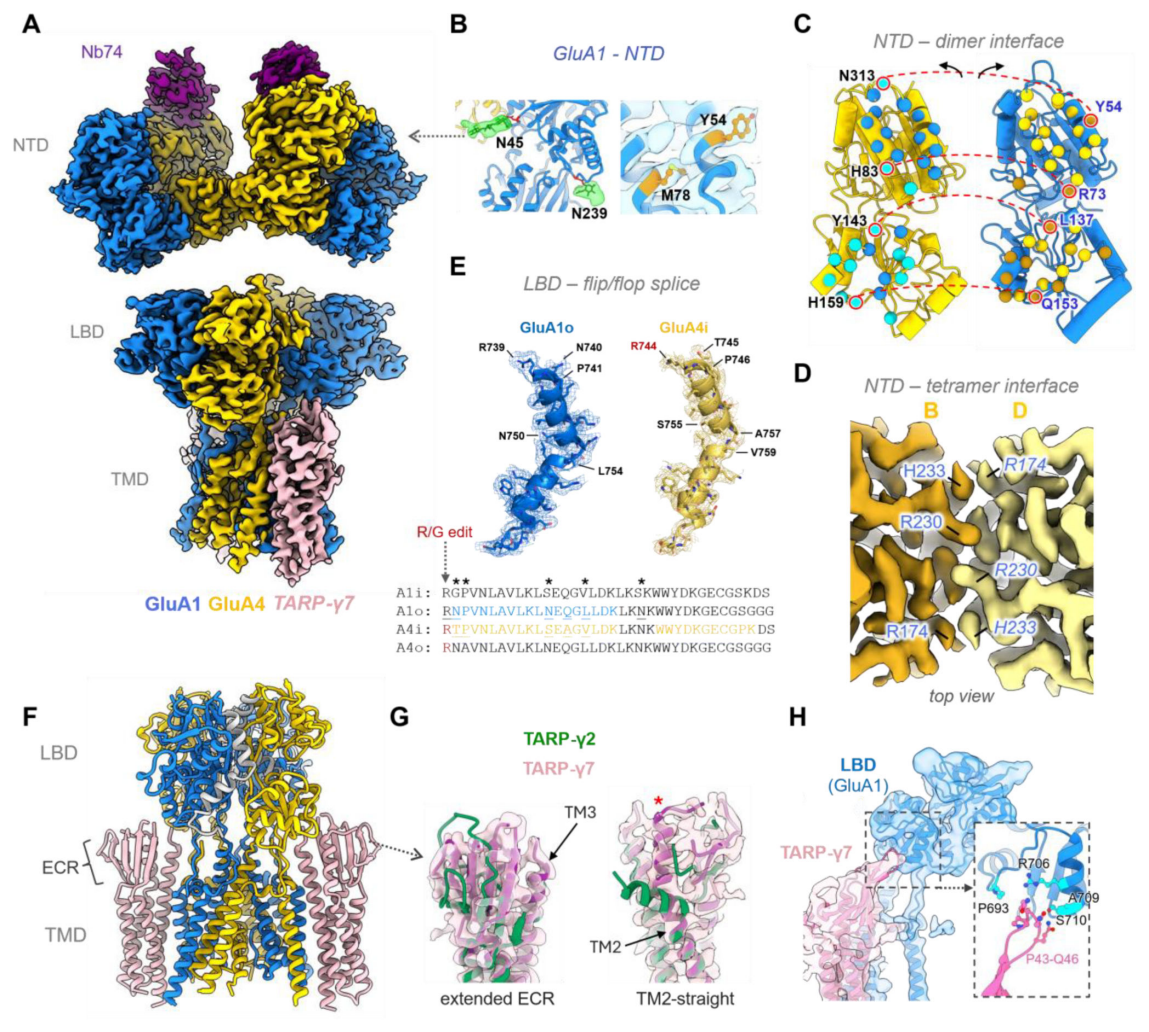
Structure of the CP GluA1/4 BG receptor. (**A**) Cryo-EM map of the GluA1/4 hexamer; subunit color-codes shown beneath. (**B**) Unique features of the A1 NTD include N-glycans at N45 and N239, as well as buried inter-dimer contacts essential for A4/A1 NTD dimer formation (Y54, M78). (**C**) ‘Open book’ of the NTD dimer interface. Selected, non-conserved (cyan and orange) GluA1 and GluA4 dimer contacts connected by red, dashed lines; conserved contact residues shown in blue and yellow. (**D**) Top view of the GluA4 tetrameric interface, key residues form a largely hydrophilic core around His210 flanked on both sides by an interfacial His233-Arg174 cation-π bridge. (**E**) Alternative splicing of GluA4/A1 identified by cryo-EM densities and MS peptides. GluA4 is unedited at the R/G site, and carries the flip exon. GluA1 harbors the flop exon. Bottom: sequence alignment highlighting the alternative residues indicated in the structure. MS peptides are highlighted in color. (**F**) LBD-TMD model of the GluA4/A1/y7 complex; color-code follows panel a. TARP-γ7 (and γ5) have unique structural properties in their extended TM3 helix, straight TM2 helix and extracellular beta-sheet which interacts with the LBD. (**G**) Comparison of the extracellular beta sheet between Type-I (γ2) and Type-II (γ7) TARPs. The TARP in the A4/A1 hexamer closely matches that of a Type-II. The non-kinked TM2 helix is indicated in the right panel (red asterisk). (**H**) Contacts between the TARP-γ7 ECR loop and the GluA1 LBD. The extensive beta-sheet projects residues Pro43-Gln46 (pink), toward Pro693 and Ala709 of the GluA1 LBD (blue).

Local refinement of the NTDs resulted in an overall resolution of ~ 3.2 Å (Fig. 2A, Table S1, and fig. S7), with both A/C subunits now clearly marked by GluA1-specific N-glycans at Asn45 and Asn239 (Fig. 2B, left panel). The GluA1/A4 NTDs match the ‘displaced’ dimer conformation generally observed with AMPAR NTDs (both homo- and heteromers, except GluA3 homomers (43); fig. S8A), and harbor an extensive dimer interface (1,600 Å^2^) (Fig. 2C). Interface contacts vary between AMPAR subunits, yielding dimers of different affinity that govern receptor biogenesis (44, 45). Affinity determinants largely locate to the sequence-conserved upper part of the interface (46), which contain two GluA1-specific residues Tyr54 and Met78 in helices B and C (Fig. 2B, right panel), both of which critically impact NTD dimer stability (44), and the GluA4-specific His83 (Fig. 2C). In addition, a hetero-dimer-specific network of interactions seals the less conserved lower part, including polar interactions by GluA1-specific residue Gln153 with GluA4 residue His159 (fig. S8B). Together, these differences will determine the greater affinity of the GluA1/A4 NTD heterodimer than the GluA4 homodimer, and dictate the dominance of GluA1/A4 heteromer formation. The NTD structure thus offers a molecular explanation for the prevalence of the GluA1/A4 subunit combination in BGs.

The NTD dimers assemble into a tetramer mediated by the inner GluA4 B/D subunits (Fig. 2A and D). This arrangement resembles the cognate interface in GluA2 (47), both in sequence and structure, with a largely hydrophilic core around His210 flanked on both sides by an interfacial His233-Arg174 cation-π bridge. The equivalent linkage in GluA2 is critical for receptor gating and synaptic transmission (14, 48, 49). Unique to GluA4 is an additional H-bond between Arg230 and Ser206 across the interface, further stabilizing the receptor. The synaptic signaling function of this arrangement is described below.

#### The GluA1/4 LBD is a flip/flop heteromer

In AMPARs, the LBD dimer interface is modified by alternative splicing (flip/flop) and RNA editing (at the R/G site), which shape gating kinetics and subunit assembly (50–55). These features are visible in our structure: GluA4 is unedited (encodes an Arg) and carries the alternative flip exon (GluA4i) (Fig. 2E and F). Supported by the MS data, we assign the flop exon to GluA1 (GluA1o) (Fig. 2E). Hence, the GluA1/A4 LBDs form an unedited flip/flop heterodimer, endowing it with a unique kinetic profile. The preferential assembly of such ‘splice-heteromers’ had been proposed (50), and this prediction is borne out in the cerebellar GluA1/A4 receptor. GluA4 Arg744 at the R/G editing site projects across the interface towards the respective GluA1 Arg739 (constitutively expressed in GluA1). This Arg-bridge is a hallmark of unedited AMPARs, seen also in GluA1 homomers, in R/G-unedited GluA2, and in recombinant GluA4 (38, 49, 52).

#### GluA1/4 associates with Type-II TARPs

**Fig. 3:**
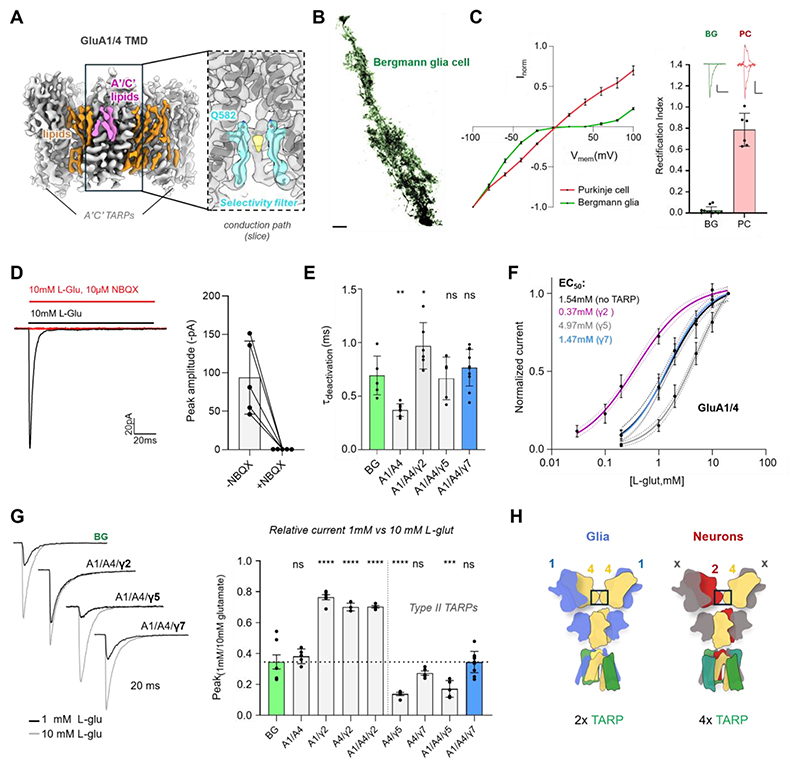
Functional characterization of the BG AMPAR. (**A**) Cryo-EM map of the GluA1/A4 ion channel sector; annular lipids are shown in orange, pink lipids populate the ‘TARP’-free A’C’ binding sites. The inset shows a cross section of the ion conduction path, with the cation selectivity filter formed by the GluA1 subunits in cyan; the yellow density at Q582 correspond to permeating ions (**B**) Confocal image of a biocytin-filled Bergmann glia cell in parasagittal cerebellar acute slice. Scale bar = 10 μm. (**C**) I/V curve of currents recorded from outside-out patches excised from either PCs or BG from cerebellar acute slice. (**D**) Outside-out recordings from BG following 100 ms 10 mM glutamate pulse without (peak 93.9±47.6 pA (mean±s.d.); n=5) or with (0.59±0.14 pA; n=5) 10 μM NBQX. (**E**) Bar plot showing the deactivation time constant measured from outside-out patches from BG cells (n = 6) and HEK293T cells expressing AMPAR/TARP complexes: GluA1/GluA4 (n = 9), GluA1/GluA4 γ2 (n = 6), GluA1/GluA4 γ5 (n = 5) and GluA1/GluA4/γ7 (n = 11). One-way ANOVA (F(_4, 32_) = 13.22, P < 0.0001), followed by Dunnett’s multiple comparisons test: *P = 0.0232, **P= 0.0028, ns = not significant; compared to BG. Bars indicate mean ± s.d., horizontal dotted line marks BG mean. (**F**) L-glutamate dose-response analysis of GluA1/GluA4 receptors alone or co-expressed with TARPs gave EC_50_ values of 1.54 mM (95% CI: 1.262 to 2.023 mM) for untarped receptors (black line), 0.37 mM (95% CI: 0.31 to 0.46 mM) for γ2 (purple line), 1.47 mM (95% CI: 1.22 to 1.88 mM) for γ7 (blue line), and 4.97 mM (95% CI: 3.98 to 6.86 mM) for γ5 (grey line). Dotted lines present 95% confidence intervals of the fits. (**G**) Left, current traces showing responses to 1- and 10-mM glutamate to the same patch excised from BG and AMPAR reconstituted in HEK cells with TARPs γ2, γ5 and γ7. Right, bar plot showing the relative 1 mM to 10 mM peak current ratio measured from outside-out patches from BG cells (n = 7) and HEK293T cells expressing AMPAR/TARP complexes: GluA1/GluA4 (n = 5), GluA1/γ2 (n = 6), GluA4/γ2 (n = 4), GluA1/GluA4 γ2 (n = 4), GluA4/γ5 (n = 5), GluA4/γ7 (n = 5), GluA1/GluA4/γ5 (n = 5) and GluA1/GluA4/γ7 (n = 11). One-way ANOVA (F_(8, 43)_ = 69.92, P < 0.0001), followed by Dunnett’s multiple comparisons test: ****P < 0.0001, ***P=0.0002, ns = not significant; compared to BG. Bars indicate mean ± s.d., horizontal dotted line marks BG mean. (**H**) Schematic of the major hexameric (left), and octameric (right) cerebellar AMPARs. The glia-derived GluA1/A4 hexamer harbors two Type-II TARPs, while the neuronal receptor contains inner GluA2 and GluA4 subunits and associates with four Type-I TARPs.

As signal intensity for the two B’/D’ TARPs varied, we combined classes with strong TARP features, and followed a processing scheme outlined in fig. S6C. The resulting TARPs exhibited three characteristics of atypical Type-II TARPs, γ5 and γ7 (Fig. 2F-H): i) an extended extracellular beta-sheet (39) bending towards the GluA1 subunit LBDs, including defined interaction points (Fig. 2H); ii) a largely un-kinked transmembrane helix 2 (TM2); and iii) an elongated TM3 in the periphery of the channel gate (Fig. 2G and figs. S4D and S8C). These features will contribute to the unique modulatory actions of Type-II TARPs (56–58). The kink in TM2 is apparent in all Type-I TARPs, is lacking in γ5 and is ill defined in γ7-AlphaFold models (fig. S8C) (59). This feature will impact positioning of the TARP extracellular region (ECR) relative to the receptor core to modulate signaling. When subjected to all-atom molecular dynamics simulations (Methods), we see that a kinked γ7 starting model readily adopts the γ5-like, straight helical conformation in seven independent simulations (usually within 5 ns), which remains throughout the 100 ns and 200 ns simulations (fig. S8D, and movie S1). This is not apparent when simulating γ2, which stays in its kinked TM2 starting conformation for 100 ns in three independent simulations (movie S2). Hence, an upright TM2 appears to be the preferred conformation of Type-2 TARPs, the function of which remains to be elucidated. Moreover, a lack of LY-481 density marking TARP-γ8 (42) led us to conclude that the GluA1/A4 hexamer preferentially harbors two Type-II TARPs at its B’/D’ sites. The unoccupied A’/C’ positions are instead decorated with annular lipids (Fig. 3A; pink densities), and the dimensions of the ion conduction path closely resembles that of the CP-GluA1 receptor (fig. S8E) (49). TARPs γ5 and γ7 are less powerful modulators than the Type-I TARPs of cerebellar octamers (7, 56–58), and both are expressed abundantly in BG (17), where they will contribute to a unique AMPAR calcium signal.

### Functional reconstitution of the BG AMPAR

BGs strictly rely on CP AMPARs for their multiple functions (22). Blocking AMPAR calcium conduction, or genetic deletion of GluA1 and GluA4 leads to the retraction of BG processes from PC synapses, accompanied by altered synaptic signaling and an excitotoxic glutamate accumulation (23, 24). Our data so far reveal a specific GluA1/4 receptor subtype, including its RNA editing and alternative splicing pattern, and modulation by Type-II TARPs. To investigate whether this subunit configuration (Fig. 2A) indeed represents the native BG AMPAR, we assessed BG responses in cerebellar brain slices using patch clamp recordings and compared them to defined AMPAR/TARP combinations expressed in HEK293 cells.

Glutamate application to patches excised from mouse BG (Fig. 3B), elicited inwardly rectifying currents, consistent with GluA2-lacking CP AMPARs, while PC patches responded with a linear current-voltage relationship, characteristic of GluA2-containing receptors (Fig. 3C) (8, 60). These BG currents were AMPAR-mediated; they were abolished by the antagonist NBQX, but were unaffected by a kainate receptor blocker (Fig. 3D and fig. S9A). Moreover, desensitization was attenuated by the positive allosteric modulator cyclothiazide (CTZ), consistent with expression of the GluA4 flip variant (fig. S9B) (60). When expressed in HEK293 cells, the heteromeric GluA1o/A4i subunit combination most closely matched the kinetics and pharmacology of native BG AMPARs (fig. S9C, D and Table S2), with GluA1o/A4i heteromer-formation confirmed by their CTZ profile (fig. S9E). To assign TARPs, we ruled out TARP-γ8, as BG responses were unaffected by the γ8-selective negative allosteric modulator JNJ-118 (fig. S9F) (61). We also eliminated TARP-γ2, as its positive modulation of gating kinetics, glutamate efficacy, and response to the partial agonist kainate did not match BG AMPARs (fig. S9D and Table S2). BG deactivation kinetics were clearly slower than those of unTARPed GluA1/4 heteromers, suggesting TARP association (Fig. 3E). Taken together, all evidence pointed toward γ5 or γ7 as the likely TARPs in BG. While both slow deactivation and enhance AMPAR conductance (fig. S9G, H and Table S2), they can be differentiated by their distinct sensitivity to glutamate.

BG sense and clear extra-synaptic glutamate to support precise PC synaptic transmission (22). The L-glutamate EC50 of BG AMPARs (~1.8 mM) (62), was not replicated by GluA1o/A4i receptors associated with γ2 or γ5 (0.37 mM and 4.97 mM, respectively). As TARP-γ7 does not impact the EC50 for glutamate (57), the presence of this TARP most closely matched BG responses (~1.5 mM) (Fig. 3F). Moreover, when comparing peak responses to 1 mM versus 10 mM glutamate, γ7-containing receptors produced a 34% peak ratio, again closely matching the 35% observed in BG AMPARs (Fig. 3G); GluA1/A4 receptors harboring γ2 or γ5 deviated markedly (70% and 17%, respectively). Therefore, CP GluA1/A4 heteromers, with a unique RNA editing and splicing pattern, are primarily modulated by two TARP-γ7 subunits to fulfil their specialized signaling function in BG (Fig. 3H) (23, 24).

### The GluA4 NTD impacts gating and synaptic transmission

A structural hallmark shared across GluA4 AMPARs is a compact, stable NTD tier (Fig. 1F, I, and fig. S2A), formed through either a homophilic (A4/A4) or heterophilic (A2/A4) interface. We next investigated the function of this conserved feature for GluA4 gating and signaling at a model synapse (facilitating a direct comparison with GluA1-3) (15, 43, 49).

**Fig. 4:**
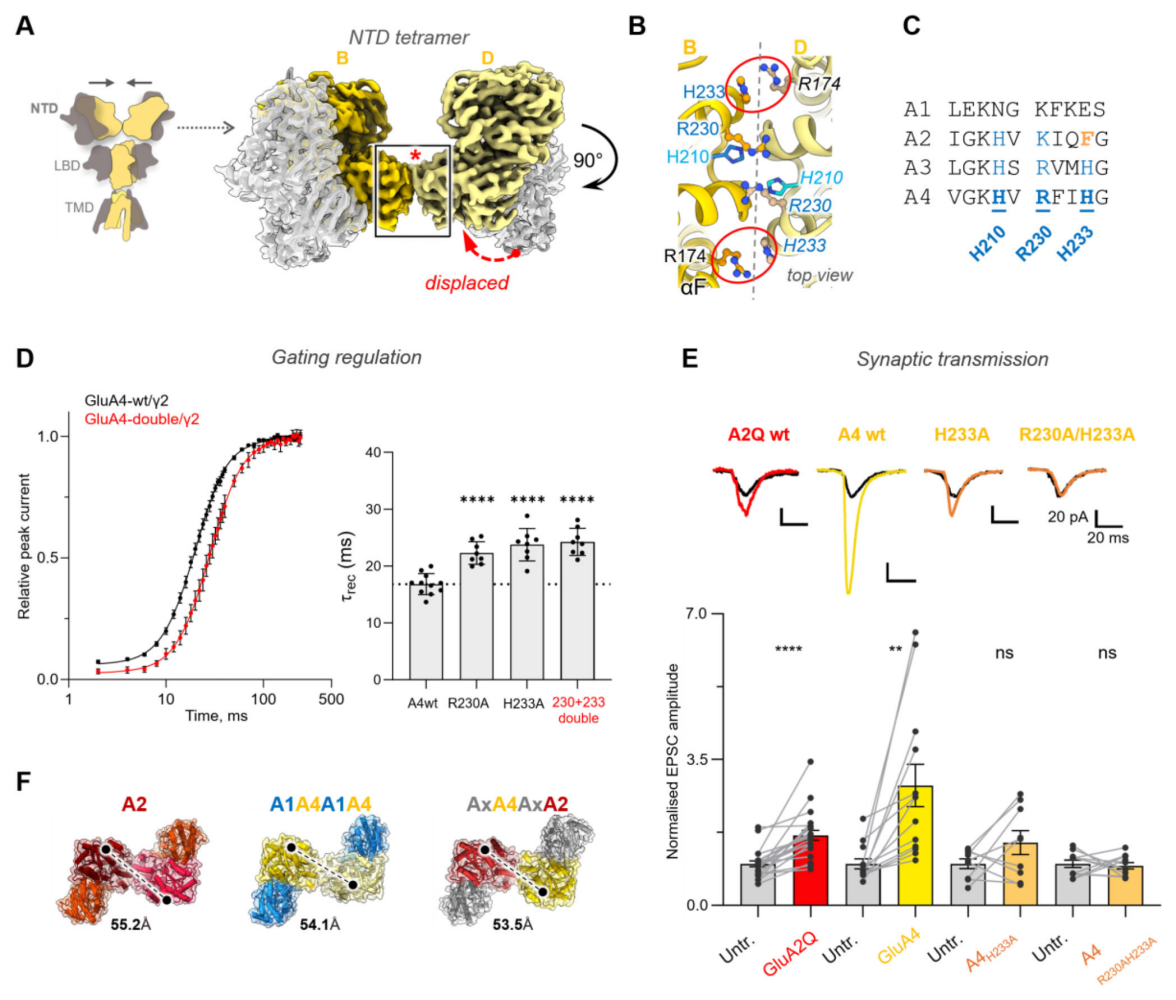
The GluA4 NTD is a strong synaptic anchor. (**A**) Schematic of an AMPAR with GluA4 B/D subunits (yellow). Cryo-EM map of the NTD tier, highlighting the tetrameric interface (boxed) between the B/D GluA4 subunits (yellow). The protomers within a NTD dimer are displaced relative to one another (red arrow). (**B**) Top view onto the B/D interface, with key interacting residues shown in stick. The Arg174-His233 cation-pi bridges are denoted with red ellipsoids. (**C**) GluA1-4 sequence alignment of the NTD interface core. (**D**) Recovery from desensitization from pooled data: GluA4/TARP-γ2 (n = 11 cells, τ = 17.1 ms) and GluA4_R230A/H233A_/TARP-γ2 (n = 8 cells, τ = 24.3 ms), both fitted with a two-component Hodgkin-Huxley equation (black line: GluA4 fit; red line: R230A/H233A fit). The slope of the fast and slow components was fixed at 4 and 1, respectively. GluA4 was edited at the R743G site. Right panel: bar graph showing recovery from desensitization following 10 mM glutamate pulses (200 ms) in cells expressing GluA4/TARP-γ2 (n = 11), GluA4R230A (n = 8), GluA4_H233A_ (n = 8), and GluA4_R230A/H233A_ (n = 8). Bars represent mean values; whiskers indicate standard deviation. One-way ANOVA (F_(3, 31)_ = 22.64, P < 0.001), followed by Tukey’s multiple comparison test. ****P < 0.0001. (**E**) Dual synaptic recordings from organotypic hippocampal slices electroporated with AMPAR constructs. EPSC amplitude is normalized to that of a neighboring untransfected cell. Paired t-test of normalized EPSC amplitude increase upon AMPAR expression. **P = 0.0026, ****P < 0.0001. Representative traces above from untransfected (grey) and transfected cells. Scale bars = 20 pA, 20 ms. (**F**) Top view onto the NTD anchor, with the NTD dimers similarly spaced between the GluA2 homomer (PDB 9B68) and the GluA4 heteromers described herein (spacing in Å is between αA of the B/D subunits).

The ordered NTDs of GluA2 and GluA4 contrast with the splayed NTDs of GluA1 and GluA3 (43, 49). This distinction, critical for synaptic function (14), arises from NTD sequence and structural features: GluA2/A3/A4 share interface-forming residues that are absent in GluA1 (Fig. 4A-C), whereas in GluA3 their involvement is sterically hindered by its atypical ‘flat’ NTD dimer configuration (43). The displaced dimer arrangement in GluA2- and GluA4-containing receptors enables NTD tetramerization between the B and D subunits (red arrow in Fig. 4A, and fig. S8A), which is established by cation-π links between an arginine and either a phenylalanine (Phe231 in GluA2) or a histidine (His233 in GluA4) (red ellipsoids in Fig. 4B). In GluA4, further stabilization is provided by Arg230, engaging Glu202 and Ser206 across the interface. The overall polar nature of the interface is underscored by solvent densities apparent at ~ 2.6 Å resolution, extending above (at Arg230) and below the contact region (figs. S7 and S10C-E).

In GluA2, NTD stability is coupled to gating and synaptic transmission (14). NTD dimer splaying, caused by mutation of Phe231 (F231A) or protonation of His208, slows GluA2 recovery from desensitization (48, 49, 63), providing a functional readout for interface stability. GluA4 exhibits a similar, albeit attenuated, dependence on its NTD for gating. Mutation of interface residues Arg230 or His233 (R230A, H233A) slows desensitization recovery, with no further reduction observed in the double mutant (R230A/H233A) (Fig. 4D). Moreover, whereas GluA2-F231A slows recovery by 2-fold (48), the equivalent GluA4-H233A mutation produces only a 1.4-fold difference. GluA4 gating is also pH-sensitive, but unlike GluA2, the NTD plays a lesser role (fig. S10F). Taken together, the GluA4 NTDs form a stable tetrameric interface, whose mutation only subtly contributes to gating of recombinant GluA4.

We then tested whether the compact GluA4 NTD arrangement impacts synaptic transmission, as observed with GluA2. Unlike GluA1 and GluA3, GluA2 expression increases excitatory post-synaptic currents (EPSCs) at CA1 pyramidal synapses, relative to untransfected neurons (13, 15, 64), presumably by anchoring the receptor close to glutamate release sites (14, 65, 66). NTD interface rupture prevents this anchorage, leading to increased GluA2 diffusion and reduced synaptic transmission (14, 49, 64). GluA4 expression in CA1 neurons also produces a substantial increase in transmission, greater than GluA2 (2.88 ± 1.8 vs 1.68 ± 0.6-fold in GluA2), likely stemming from its stable NTD tier. Indeed, destabilization by the H233A mutation (the GluA2 F231A equivalent) abolished the increase in transmission, which was decreased further with the GluA4 double mutant (H233A/R230A) (Fig. 4E). Both mutants lowered the synaptic rectification index similar to GluA4 wt, suggesting comparable expression of homomeric GluA4 at the synapse (fig. S10G) (67). Hence, GluA4 efficiently anchors at CA1 synapses like GluA2, and in stark contrast to both GluA1 or GluA3 (43, 49), supporting the existence of two AMPAR subgroups, that can be segregated by their NTD architecture (fig. S10H).

As the GluA2 and GluA4 NTDs share only ~60% sequence identity, the synaptic anchoring machinery appears to recognize spatial features of the tetrameric assembly. This is supported by the comparable spacing between the NTD dimers in GluA2 and in CP and CI GluA4 heteromers (Fig. 4F). A recent study implicated noelin-1 (noe-1), a secreted synaptic anchoring protein (68), in docking to GluA4 B/D subunits (69). Noe-1 knock-out resulted in a reduction of spontaneous EPSCs in CA1 (68). Whether noe-1 contributes to the increased EPSCs observed with GluA2 and GluA4 at CA1 synapses needs to be investigated in further studies.

## Discussion

Taken together, structurally and stoichiometrically distinct GluA4 AMPAR-TARP complexes perform the specialized functions of cerebellar neurons and glia (Fig. 3H). Group 1 receptors incorporate GluA2 into one of the gating-dominant B/D positions, with yet to be defined subunits at the A/C sites further tuning calcium influx (8), and four Type-I TARPs as powerful allosteric modulators (movie S3). These receptors will largely derive from the highly abundant GC neurons, and are expected to dominate transmission at the MF-GC synapse, forming a major afferent input into the cerebellum (Fig. 1A and J) (20, 70, 71). Whereas, group 2 receptors are CP GluA1/A4 heteromers of defined arrangement (Fig. 2A and movie S4), primarily derived from glia. These receptors associate with two Type-II TARPs, predominantly γ7 (Figs. 2F-H, and fig. S1I), which alter glutamate affinity to shape calcium signaling optimized for BG function (Fig. 3D) (22, 72).

The robust synaptic delivery of GluA2 will support its canonical ‘housekeeping’ role as a Na^+^-conducting glutamate receptor, enabling reliable baseline excitatory transmission across principal neurons of the brain. By contrast, GluA4 is calcium-permeable, and unlike the CP GluA1 and GluA3 subtypes (43, 49), robustly traffics to synapses (Fig. 4E). Moreover, GluA4 competes with GluA2 for the gating-dominant B/D subunit positions (Fig. 1F, H and S10A), effectively ‘diluting’ GluA2’s contribution, thereby facilitating calcium conduction. AMPAR calcium influx can be highly excitotoxic (1, 73), and is a possible outcome of the potent synaptic trafficking of GluA4. However, GluA4 expression is under tight developmental control; in the CA1 region, GluA4 is replaced by GluA2-containing AMPARs upon circuit maturation (74). GluA4 also differs from the other AMPARs in its speed of gating (54), and its highly restricted expression pattern: in the mature forebrain, GluA4 is mostly confined to inhibitory interneurons (75), and the cerebellum. These spatial and temporal constraints may dampen the excitatory drive of this otherwise powerful glutamate receptor. In the cerebellum, the specific organization of GluA4 AMPARs mediates diverse signaling roles tailored to the unique demands of neurons and glia (Fig. 1A and 3F,H).

## Materials and Methods

### Production and purification of Nb74

To produce Nb74 in bacterial cells, the Nb74-HIS-CSC/pMESy4 vector (ARA; *to be published*) was transformed into WK6K-Su cells by heat shock. Cells were then pre-cultured in 100 μl of SOC medium at 37°C for 1 hour, followed by a second pre-culture in 20 ml of LB broth with 2% glucose, 1 mM MgCl2 and ampicillin overnight. 10 ml of the preculture was then added into 500 ml Terrific Broth with 0.1% glucose, 2 mM MgCl2, and ampicillin, and incubated while shaking at 37°C until the OD600 reached 0.6-0.7. Protein production was induced by IPTG (1 mM final concentration) and cultures were incubated at 28°C while shaking overnight (15-16 hours). The pellets from overnight culture were collected by centrifugation at 5000 x g for 30 min and then exposed to a sucrose osmotic shock upon resuspension. The resuspension was then incubated at 4°C for 1-2 hours in 12.5 ml of TES buffer (20 mM Tris-HCl pH 8.0, 0.5 mM EDTA, 0.5 M sucrose) to release nanobodies from the periplasmic space. The supernatant was then collected by the centrifugation at 15,000 x g for 30 min and then incubated with Ni-NTA beads. After binding, beads were washed with washing buffer (20 mM Tris-HCl pH 8.0, 500 mM NaCl) and washing buffer containing 10 mM imidazole. Protein was finally eluted by incubation with 0.1 mg/ml 3C protease for 10 hours at 4°C. The final elution was filtered with 0.22 um PEI filter (Millipore). The filtered elution was subjected to gel filtration on a Superdex 200 increase 10/300 GL column in washing buffer (20 mM Tris-HCl pH 7.4, 500 mM NaCl), and the peak fractions were collected and concentrated on 10kDa cut off filters (Amicon, Millipore).

For mammalian cell production, Nb74 was cloned into the pHLsec-Fc vector and produced by transient transfection in expi293F cells (76). 44-48 hours post transfection, cells were harvested at 6,000 x g (JLA 8.1000) and the resultant supernatant was filtered using a 0.22 µm filter. Filtered supernatant containing secreted Nb74-Fc was exchanged into buffer containing 20 mM Tris-HCl pH 8.0, 150 mM NaCl, and then concentrated to a volume of ~100 ml (10 kDa filter cut-off) using the AKTA FluxS crossflow filtration system. The buffer-exchanged supernatant was incubated with Ni-NTA agarose beads for 2 hours at 4°C. Nb74-bound beads were washed sequentially with washing buffer (20 mM Tris-HCl pH 8.0, 150 mM NaCl,) containing 20 mM, 40 mM, and 80 mM Imidazole. Elution was done in a buffer containing 20 mM Tris-HCl pH 8.0, 150 mM NaCl, 300 mM Imidazole. The purified Nb74-Fc was dialyzed overnight into imidazole-free buffer (20 mM Tris-HCl pH 8.0, 150 mM NaCl) using SnakeSkin™ Dialysis Tubing (10 kDa cut-off), and stored in 2 ml aliquots (~ 1 mg/ml) at -80°C.

### Production of biotinylated GluA4-NTD

The GluA4-NTD was cloned into the pHLsec-Avitag3 vector (76) and transiently transfected into HEK293T cells expressing an ER-resident BirA variant (BirA-ER) (77). After 72 hours of culture in the presence of 100 μM D-biotin, conditioned media was collected, dialyzed, aliquoted and stored at -80°C.

### Surface Plasmon Resonance

SPR experiments were performed on a Biacore T200 instrument (Cytiva) operated at 25 °C and at a data collection frequency of 10 Hz. The running buffer was HBS-CT (20 mM HEPES pH 7.4, 150 mM NaCl, 3 mM CaCl2, and 0.005% (v/v) Tween-20) supplemented with 1.0 g/L bovine serum albumin. Biotinylated GluA1/2/3/4-NTD proteins (immobilized ligand) were captured to 3159, 2093, 5427, and 1000 RU respectively. A reference channel immobilized with biotinylated CLNB except for GluA4-NTD where GluA2-NTD was used. Purified NB74 protein (analyte) aliquots were thawed and diluted to 100 nM concentration using the SPR running buffer. Single cycle kinetic assays were performed by injection of 5 successive concentrations of the analyte, prepared in a three-fold dilution series from the 100 nM stock, in order of increasing concentration. Each sample was injected for 120 s at a flow rate of 30 µL/min. The final analyte injection was followed by a 900 s dissociation phase. Fitting and analysis of the kinetic binding data (1:1 Langmuir binding mode) were performed using the Biacore SPR Analysis software (Cytiva).

### Purification of native GluA4 AMPAR complexes

AMPARs were purified natively from freshly culled pig brains (> 5 months old). Fresh brains were acquired from a butcher and transported in ice-cold PBS, cleaned in homogenization buffer (5 mM HEPES pH 8.0, 320 mM d-sucrose, cOmplete EDTA-free protease inhibitors), and the cerebellum was dissected with a clean blade by cutting along the junction to the cerebral cortex, before being immediately homogenized in ice-cold buffer with a low-clearance teflon-glass homogenizer. Debris was cleared by centrifugation at 1,000 x g for 10 minutes at 4°C, and the resulting supernatant (S1) was centrifuged at 12,000 x g for 20 min at 4°C (Sorvall SS-34). The synaptosome pellet (P2) was collected, resuspended in homogenization buffer, and snap-frozen in liquid nitrogen and stored at -80°C. Synaptosome pellets were thawed on ice and resuspended in ice-cold water (10:1) and hypo-tonically lysed for 15 minutes at 4°C under gentle rotation. The resuspension was then centrifuged at 25,000 x g for 30 min at 4°C (Sorvall SS-34). The resulting pellet (P2’) was then lysed in lysis buffer (20 mM Tris-HCl pH 8.0, 150 mM NaCl, 5 μM NBQX, cOmplete EDTA-free protease inhibitors) with either 1% digitonin (w/v) or 1%/0.1% LMNG/CHS (w/v) for 3 hours at 4°C under gentle rotation. Insoluble material was then removed by ultra-centrifugation at 41,000 rpm for 45 minutes at 4°C (Beckman 45Ti), and the resulting supernatant was aspirated on ice. The lysate was pre-cleared with protein-A agarose bead slurry (sc-2001) for 1 hour at 4°C. For GluA2 depleted samples, 120 μg of the GluA2 CTD antibody (mouse monoclonal against epitope CVAKNAQNINPSSSQ produced by GenScript) was added during the pre-clearing step, before subsequent binding with 2 mg Nb74-Fc at 4°C. After binding with protein-A agarose bead slurry, bound fractions were washed in 3 column volumes of GDN buffer (20 mM Tris-HCl pH 8.0, 150 mM NaCl, 0.05% GDN). The protein was eluted from the Fc-bound Protein-A beads by digestion with 3C protease overnight at 4°C. Eluted fractions were pooled and concentrated to 2–3 mg/ml for cryo-EM grid preparation.

### Purification of recombinant GluA4-TARP γ2 complex

For expression and purification of GluA4 homomers with TARP γ2, constructs of eGFP-tagged GluA4 (pRK5) and TARP γ2 (pRK5) were co-transfected into expi293F cells at a ratio of 1:1. AMPAR antagonists ZK200775 (2 nM, Tocris, 2345) and kynurenic acid (0.1 mM, Sigma, K335-5G) were added to the culture medium to prevent excitotoxicity. 44-48 hours post transfection, cells were harvested at 3,000 x g (JLA 8.1000) and the resultant pellet was washed with ice-cold phosphate-buffered saline (PBS) to removed residual medium. Cells were lysed in lysis buffer (20 mM Tris-HCl pH 8.0, 150 mM NaCl, 5 μM NBQX, cOmplete EDTA-free protease inhibitors) with 1% digitonin (w/v) for 3 hours at 4°C under gentle rotation. Insoluble material was then removed by ultra-centrifugation at 41,000 rpm for 45 minutes at 4°C (Beckman 45Ti), and the resulting supernatant was pre-cleared with protein-A agarose bead slurry (sc-2001) for 1 hour at 4°C. For pulling down the GluA4 homomeric complexes, 2 mg of Nb74-Fc was used. After binding with protein-A agarose bead slurry, bound fractions were washed in 3 column volumes of GDN buffer (20 mM Tris-HCl pH 8.0, 150 mM NaCl, 0.05% GDN). The protein was eluted from the Fc-bound Protein-A beads by digestion with 3C protease overnight at 4°C. Eluted fractions were pooled and concentrated to 2–3 mg/ml for cryo-EM grid preparation.

### Cryo-EM grid preparation and data collection

Cryo-EM grids were prepared using a FEI Vitrobot Mark IV. To capture the cerebellar A4 proteome and recombinant GluA4-TARP γ2 in a resting state, protein was incubated with 200 μM NBQX for at least 30 minutes on ice before freezing. For native GluA2 depleted dataset, protein was incubated with 200 μM NBQX and 40 μM LY-481 for at least 30 minutes on ice before freezing. The TARP γ8 specific ligand LY-481 was applied 30 minutes prior to freezing to improve clarity on the auxiliary subunits. Quantifoil holey carbon grids (300 mesh Au) were glow discharged in a PELCO easiGlow™ glow discharge apparatus at 25 mA for 30s, prior to sample application. 4 μl sample was applied to the grids, blotted for 2.5-3.5 seconds at 4°C with 100% humidity and plunge-frozen in liquid ethane.

For the GluA2 containing native GluA4 and the recombinant GluA4-TARP γ2 datasets, data were acquired using EPU3 (AFIS enabled) on a Thermo Fisher Scientific (TFS) 300 keV Titan Krios equipped with Falcon4i detector (TFS) and TFS Selectris X energy filter operating at 10 eV slit width. Data collection was performed at a magnification of 130 kx in counted super-resolution mode with 2× binning, resulting in a pixel size of 0.955 Å per pixel. Movies were recorded for 40 frames and 3.51 s resulting in a total dose of 40 e^−^/Å^2^. The defocus values ranged from −1.2 to −2.4 µm. For the native GluA2 depleted dataset, data were acquired using EPU3 (AFIS enabled) on a Thermo Fisher Scientific (TFS) 300 keV Titan Krios equipped with K3 detector (Gatan) and GIF quantum energy filter operating at 20 eV slit width. Data collection was performed at a magnification of 105 kx in counted super-resolution mode with 2× binning, resulting in a pixel size of 0.826 Å per pixel. Movies were recorded for 40 frames and 1.8 s resulting in a total dose of 40 e^−^/Å^2^. The defocus values ranged from −1.2 to −2.4 µm.

### Cryo-EM data processing and model building

For the GluA2 containing dataset, a total of 51,245 movies were imported into RELION 5.0 (78), and beam-induced motion was corrected using MotionCor2 (79). Motion corrected micrographs were imported into cryoSPARC v4.6.0 (80) for cleaning the particle stack. Contrast transfer function was estimated by patch CTF estimation. Blob picker was used to pick particles from around 2,000 micrographs to generate 2D class averages for template-based particle picking and *ab initio* reconstruction. Here, 2D class averages with clear receptor features were used as a template to pick the entire dataset. Meanwhile, two *ab initio* reconstructions were run. The first one was used to generate an initial AMPAR model. The same job was cloned and killed after the first iteration to generate noise models. Particles picked from all the micrographs were inspected and then extracted with a binning factor of 4 at a box size of 128 × 128 pixels. The extracted particles were subjected to several rounds of heterogeneous refinement using 4-5 noise models and the initial *ab initio* reconstruction to clean the particle stack. Good particles were scaled to a binning factor of 2 and used as an input for Non-Uniform Refinement with dynamic masking. The resulting 521,937 refined particles were re-imported into RELION 5.0 using the python script csparc2star.py (https://doi.org/10.5281/zenodo.3576630). To address heterogeneity in the dataset, the refined particle stack was 3D classified (no mask) into 10 classes. Receptors were grouped into two prominent groups based on the TARP occupancy – group 1 containing 4 TARPs and group 2 containing 2 TARPs. A subpopulation of group 2 which showed flexible NTDs were further classified (with alignment) into 5 classes giving rise to a small group of splayed NTDs (group 3). About 10% of the total receptors from this dataset exhibited splayed NTDs and 2 TARPs (fig. S2B).

Group 1, containing 65,211 particles, were processed using two approaches. First, we performed NTD focused classification with a loose mask (fig. S4A). NTD classes showing heterogeneous Nb74 occupancy were selected and refined full-length (C1). To better resolve the unlabeled AMPAR subunits, we scaled the particles to original pixel size and performed Non-Uniform Refinement (C1) of the full-length GluA4/GluAx receptor followed by local refinements (C2) of the individual layers (NTD, LBD, and TMD-TARP). The high-resolution NTD from the local refinement job was then symmetry expanded in RELION. The symmetry expanded particles were classified into 10 classes with a regularization parameter of 100. We selected good classes, removed particle duplicates and refined the NTD and the LBD-TMD-TARP sector (fig. S4C). To generate a high-resolution GluA2/A4 NTD interface, we pooled all the NTD classes with Nb74 at B site and un-labelled AMPAR at D site, removed duplicate particles, and refined the heterophilic NTD with no symmetry imposed (fig. S7C).

To further refine Group 2 particles containing a compact NTD and 2 TARPs, two focused 3D classification jobs were performed - one with a loose NTD mask, and the other with a loose LBD-TMD-TARP mask. This gave rise to 84,740 GluA1/A4 NTD particles and 68,031 GluA1/A4 LBD-TMD-TARP particles. The particles were scaled back to original pixel size and Non-Uniform Refinement (C1) and individual local refinements (C2) were done for these stacks to obtain a composite Group 2 map. An approach identical to the Group 1 NTD (described above) was taken to understand the Nb74 occupancy of the Group 2 NTD (fig. S5A).

For the GluA2 depleted dataset, two independent sets of purification were performed and individual datasets with 32,072 and 23,955 movies were collected. The same steps as previously described above were used to generate clean particle stacks. The datasets were processed independently and only the particles contributing to the composite structure were merged. 574,407 and 529,442 particles from the first Non-Uniform Refinement jobs were re-imported into RELION 5.0. Each dataset was 3D classified (no mask) into 10 classes. All the classes had 2 TARPs and were therefore classified as Group 2 receptors. To generate a high-resolution map, classes with the strongest signal for Nb74 and GluA1-specific N-45 glycan were pooled (329,869 particles). Particles were scaled to original pixel size and Non-Uniform refinement (C1) was performed followed by local refinements (C2) of the individual layers, giving rise to high-resolution maps for GluA1/4 NTD and LBD, respectively (fig. S7). The TARP densities however were sub-optimal, so we followed a different strategy to obtain high-resolution TMD-TARP map. No density for TARP γ8 ligand LY-481 was observed. Again, the datasets were treated independently at this stage. From the initial Non-Uniform Refined particles, only signal corresponding to the LBD-TMD-TARP sector was retained by using an inverted mask of the region (fig. S6). The particles were then 3D classified iteratively and only classes with the strongest density for TARP extracellular domain were pooled for refinement (124,708 particles). Particles were scaled to their original pixel size and refined with Non-Uniform refinement (C1) followed by local refinement (C2) of the TMD-TARP sector, which gave rise to the GluA1/A4 TARP Type 2 map (fig. S7D).

For the recombinant GluA4-TARP γ2 dataset, a total of 12,755 movies were collected and the same steps as described above were performed to obtain a clean particle stack. 293,106 particles from the first Non-Uniform refinement (C1) were classified into 5 classes without a mask. A single class (46,123 particles) showing an ordered NTD layer was taken for further refinement. The particles were scaled up to original pixel size and Non-Uniform refinement (C1) followed by individual local refinements (C1) of the NTD and LBD-TMD-TARP γ2 sectors gave rise to the Nb-74 bound homomeric GluA4-TARP γ2 map (fig. S3)

Model building and refinement for high-resolution structures were performed using Coot (81), Refmac-Servalcat (82), ISOLDE (83) and PHENIX real space refinement (84). Initial models for GluA4 and TARP γ7 were generated from AlphaFold3 (Uniprot ID I3L8N9 and P62956), while initial models for GluA1 were from PDB 7OCE (LBD and TMD) and Alphafold3 (Uniprot ID A0A286ZS63 for NTD). Individual chains were first rigid-body fit into the EM density map using ChimeraX (85), and then manual refinement was performed in Coot to further refine the geometry, and several rounds of PHENIX real-space refinement and manual refinement were performed iteratively. Data processing and model validation statistics are provided in Table S1. All final models were evaluated with MolProbity (86). Figures were prepared using UCSF ChimeraX (85), pyMOL (87) and Coot (81).

### NTD Interface Conservation Analysis

The sequences of AMPAR core subunits GluA1, GluA2, GluA3 and GluA4 from a range of vertebrates (mammals, reptiles, amphibians and fish) were downloaded from the Ensembl database (88) and the region corresponding to the NTD (residues 1-375) was extracted and aligned with MUSCLE (89) within UniPro UGENE (90). Duplicated entries and sequences with missing regions or unidentified residues were removed, as well as columns with large numbers of gaps, and the alignments were manually corrected by inspection of the 3D structures. The resulting alignment with 164 sequences was uploaded to the Consurf web server (91), along with the GluA1/A4 heteromer structure from this study. The results were visualized on the protein surface in PyMOL 2.5 using the PyMOL session file provided by the Consurf server.

### DNA constructs and cell culture

cDNAs encoding the rat GluA4 flip isoform (unedited at the R744G site) and the GluA1 flop isoform were subcloned into the pIRES vector. Mutations were performed using IVA cloning as previously described (92). TARPs γ2 and γ5 were of rat origin; TARP γ7 was derived from the human coding sequence, but the encoded protein is identical in human, rat, mouse, and pig. HEK293T cells (ATCC CRL-11268, RRID: CVCL_1926, Lot 58483269; STR-authenticated, mycoplasma-free) were maintained in DMEM (Gibco 10569010) with 10% FBS and penicillin/streptomycin at 37°C, 5% CO_2_. Cells were transfected with 1 µg total DNA using Effectene (Qiagen) or Turbofect (Thermofisher) at an AMPAR:TARP ratio of 1:2. For heteromeric recordings GluA1_flop_/GluA4_flip_ were co-transfected at 2:1 plasmid ratio since GluA1 flop does not traffic to membrane surface efficiently (51). To prevent AMPAR-mediated toxicity, 30 μM NBQX (Tocris or HelloBio) was added during transfection. Recordings were performed 24-48 h post-transfection.

### Electrophysiology

Recording pipettes (GB150F-8 0.86ID, 1.5OD with borosilicate filament, Science Products with resistance of 2-4 MΩ for whole-cell and 6-12 MΩ for outside-out patches) were pulled with a P-1000 (Sutter) and polished with MF-830 (Narishige). Internal solution contained (in mM): CsF 120, CsCl 10, EGTA 10, HEPES 10, Na_2_ATP 2, spermine 0.1 (pH 7.3, CsOH). External solution contained (in mM): NaCl 145, KCl 3, CaCl_2_ 2, MgCl_2_ 1, glucose 10, HEPES 10 (pH 7.4, NaOH). Recordings were performed with an Axopatch 700B amplifier, filtered at 10 kHz, digitized at 100 kHz (Digidata 1550B), and analyzed using pClamp 11.2.

Cells were plated on poly-L-lysine-coated coverslips on the day of the recording. Solution exchange (20–80% rise time) was ~300 µs (whole-cell) and ~120 µs (outside-out) using a theta-tube (300 µm ID) mounted on a piezoelectric translator (Physik Instrumente). The input signal to piezo amplifier was filtered at 250 Hz to reduce mechanical oscillations. Cells and patches were held at -60 mV (not corrected for 8.5 mV junction potential); series resistance was never higher than 8 MΩ and compensated 80-90% (whole cell recordings).

Desensitization time constants were obtained by fitting current decay (90% peak to baseline) with one or two exponentials (Clampfit 11.2). For biexponential fits, τ_w_,des = τ_f_(A_f_ / (A_f_ + A_s_)) + τ_s_(A_s_ / (A_f_ + A_s_)). Recovery from desensitization was measured using a two-pulse protocol (10 mM glutamate, 200 ms conditioning pulse, followed by 10 mM glutamate, 15 ms applied at various time intervals 2-40 ms and 50 to 240 ms). For pH modulation (pH 7.4 vs pH 5.5) and BG native responses a second pulse was applied in in interval of 10 ms. Recovery from desensitization data was fitted with Hodgkin-Huxley type equations ^(93)^: for outside out recovery obtained from patches the recovery profile was fitted with:

$$\text{y}={\text{y}}_{0}+\text{a}1^{*}{(1-\text{exp}\left(-{\text{x}}^{*}\text{k}\right))}^{\text{m}}$$

where k is the rate of recovery, and m is the slope which was fixed to m = 2 since the GluA4 construct has a recovery profile (94). The recovery for whole cell recordings was fitted with the function that is a sum of two Hodgkin-Huxley terms y=y_0_+ a1*(1-exp(-x*k_1_))^m1^ + (y_max_-a1-y_0_)*(1-exp(-x*k_2_))^m2^ where k_1_ and k_2_ are rates of recovery and m_1_ and m_2_ are slopes. Good fits could be obtained by fixing the slopes m_1_ and m_2_ to 4 and 1 respectively. The y_max_ was constrained to 1. The weighted tau of recovery was calculated as:

$$\tau_{\text{w}}=\left(\left({\tau_{1}}^{*}\text{a}1\right)+{\tau_{2}}^{*}\left({\text{y}}_{\text{max}}-\text{a}1-{\text{y}}_{0}\right)\right)/\left({\text{y}}_{\max}-{\text{y}}_{0}\right).$$

The glutamate dose-response relationship of GluA1/GluA4 receptors in complex with TARPs γ2, γ5, and γ7 was measured from outside-out patches at holding potential of -60 mV. In each recording, up to three glutamate concentrations were applied alongside the 20 mM glutamate, which was the highest concentration tested. The sodium concentration of all solutions was adjusted to match that of the 20 mM glutamate. A total of five glutamate concentrations were tested, and their response was normalized to the 20 mM concentration to generate the full dose-response curves. For the GluA1/GluA4 expressed together with TARPγ5, which substantially decreased glutamate potency, 20 mM may have been below the saturating concentration, and thus the reported EC_50_ value for this AMPAR/TARP complex could be an overestimate. The dose-response relationship for AMPAR/TARP complex was fitted with GraphPrism software using the Hill equation:

$$I=\frac{I_{\text{max}}{\left[A\right]}^{nH}}{{\left[A\right]}^{nH}+EC_{50}^{nH}}$$

where *I*_max_ is the maximum response, EC_50_ is the concentration of glutamate that gave half of the maximum response and *n*_H_ is the Hill coefficient.

Nonstationary fluctuation analysis (NSFA) was performed on the desensitizing current phase of macroscopic currents evoked with glutamate pulses (10 mM, 200 ms) from outside-out patches containing GluA1/GluA4, GluA1/GluA4/γ5 and GuA1/GluA4/γ7. The variance (σ^2^) of 30-80 successive responses was grouped in ten amplitude bins, plotted against the mean current, and fitted with a parabolic function:

$$\sigma^{2}=i\bar{I}-{\bar{I}}^{2}/N-\sigma_{o}^{2}$$

where *i* is the single-channel current, *I* is the mean current, *N* is the number of channels and σ_o_^2^ is the background variance. The weighted mean single-channel conductance (γ) was obtained from the single-channel current and the holding potential (−60 mV, not corrected for the liquid junction potential).

### Animals

All procedures were carried out under PPL PP5747704 in accordance with UK Home Office regulations and licensed under the UK Animals (Scientific Procedures) Act of 1986, following local ethical approval. Wild-type C57BL/6Jola (RRID: MGI:3691859) animals were housed with food and water ad libitum on a 12 h light/dark cycle at room temperature (20–22°C) and 45–65% humidity.

### Acute cerebellar slices

Mice at postnatal days 19-20 were briefly anaesthetized with 4% isoflurane in oxygen and decapitated. The brain was removed in ice-cold sucrose cutting artificial cerebrospinal fluid (aCSF) containing (in mM): sucrose (252), KCl (3), NaH2PO4 (1.25), MgSO4 (5), CaCl2 (0.1), glucose (10), NaHCO3 (26.4). Parasagittal cerebellar acute slices (250 μm thick) were cut using a vibratome (Leica VT 1200S) and left to recover for 30 min at 37°C in sucrose aCSF and for a further 1 hr at room temperature in recording aCSF containing (in mM): NaCl (126), KCl (3), NaH_2_PO_4_ (1.25), MgSO_4_ (2), CaCl_2_ (2), glucose (10), NaHCO_3_ (26.4), both saturated with 95% O_2_/5% CO_2_.

Bergmann glial cells were identified visually based on their small soma and close proximity to Purkinje cell bodies. Cell identity was confirmed during whole cell patch clamp by their low input resistance, negative resting potential (~ -80 mV) and the absence of action potentials during a current step protocol. Outside-out recordings, voltage clamped at -60 mV, were performed using borosilicate pipettes (5-9 MΩ) filled with internal solution containing (in mM): CsF (120), CsCl (10), EGTA (10), HEPES (10), Na2-ATP (2) and spermine (0.15), adjusted to pH 7.3 with CsOH. For post-fixation confirmation of BG cell type, biocytin (Sigma) was added to the intracellular solution at 4 mg/ml. The theta-tube extracellular solution contained (in mM): NaCl (145), KCl (3), CaCl_2_ (2), MgCl_2_ (1), glucose (10) and HEPES (10), adjusted to pH 7.4 using NaOH.

### Organotypic hippocampal slices

Organotypic slice cultures were prepared from hippocampi extracted from mice at postnatal day 6–8, immersed in high-sucrose Gey’s balanced salt solution containing (in mM): sucrose (175), NaCl (50), KCl (2.5), NaH_2_PO_4_ (0.85), KH_2_PO_4_ (0.66), NaHCO_3_ (2.7), MgSO_4_ (0.28), MgCl_2_ (2), CaCl_2_ (0.5) and glucose (25) at pH 7.3. Slices of 300 μm thickness were cut using a McIlwain tissue chopper and cultured on Millicell cell culture inserts (Millipore) in equilibrated slice culture medium (37 °C/5% CO_2_) containing: MEM (78.5%), heat-inactivated horse serum (15%), B27 supplement (2%), 1 M HEPES (2.5%), 0.2 M GlutaMAX supplement (1.5%), 0.05 M ascorbic acid (0.5%), CaCl_2_ (1 mM) and MgSO_4_ (1mM).

Single cells from the CA1 region of organotypic hippocampal slices were transfected at DIV 5-10. DNA plasmids were diluted to 33 ng/μl at a 1:7 ratio of pN1-eGFP to AMPAR-expressing plasmid in intracellular solution containing (in mM): KGlu (125), KCl (20), MgCl_2_ (4), HEPES (10), Na_2_-ATP (4), Na-GTP (0.3), EGTA (0.2) and back-filled into borosilicate microelectrode pipettes (5-9 MΩ). Slices were placed in the recording chamber sterilized with 70% ethanol and filled with HEPES-based aCSF containing (in mM): NaCl (140), KCl (3.5), MgCl_2_ (1), CaCl_2_ (2.5), HEPES (10), glucose (10), Na-Pyruvate (1), NaHCO_3_ (2). Cells were briefly kept in cell-attached mode and DNA was introduced with a short burst of current pulses (60 pulses at 200Hz). Slices were returned to incubation in their original culture medium supplemented with 5 μg/ml gentamycin until recording 3 days later.

Synaptic recordings were performed in aCSF containing (in mM): glucose (10), NaH_2_CO_3_ (26.4), NaCl (126), NaH_2_PO_4_ (1.25), KCl (3), MgSO_4_ (4), CaCl_2_ (4), 2-chloroadenosine (0.002), D-AP5 (0.1), and SR-95531 (0.001), and saturated with 95% O_2_/5% CO_2_. Pipettes (3–5 MΩ) were filled with intracellular solution containing (in mM): CH_3_SO_3_H (135), CsOH (135), NaCl (4), MgCl_2_ (2), HEPES (10), Na_2_-ATP (4), Na-GTP (0.4), spermine (0.15), EGTA (0.6), CaCl_2_ (0.1) at pH 7.25. EPSCs were evoked by 0.2 Hz Schaffer collateral stimulation using a monopolar glass electrode filled with aCSF, and responses were simultaneously recorded from a pair of GFP-positive and -negative cells situated in close proximity to one another. Patch clamp signals were acquired using a Multiclamp 700B amplifier, digitized by Digidata 1550B (both Axon Instruments) and recorded using pCLAMP 10 (Molecular Devices).

### TARP immunoprecipitations

Synaptosomes were solubilized in lysis buffer (20 mM Tris-HCl pH 8.0, 150 mM NaCl, 1% digitonin (w/v), cOmplete EDTA-free protease inhibitors) for 3 hours at 4°C under gentle agitation. Insoluble material was then removed by ultra-centrifugation at 41,000 rpm for 45 minutes at 4°C (Beckman TLA-55), and the resulting supernatant was aspirated on ice. The lysate was pre-cleared with protein-A agarose beads (sc-2001) for 60 minutes at 4°C, before immunoprecipitation was performed with 10μg of the following polyclonal antibody combinations in separate tubes: TARP γ2 (07-577), TARP γ5 (ACC-115) and TARP γ7 (AF-720). Immunoprecipitation was performed overnight at 4°C. After binding with protein-A agarose bead slurry, bound and unbound fractions were loaded onto 3-12% Bis-Tris SDS-PAGE gels. Proteins were then transferred to PVDF membranes and Western Blotting was performed with corresponding GluA1-4 primary antibodies and HRP-conjugated secondary antibodies.

### Semi-quantitative mass spectrometry

For mass spectrometry analysis, purified AMPAR complexes were prepared as for cryo-EM data collection, in triplicates. For the GluA2-depleted sample, 120 μg of anti-GluA2 antibody (mouse monoclonal against epitope CVAKNAQNINPSSSQ produced by GenScript) was incubated with the lysate along with the protein-A agarose beads during the pre-clearing step. For the LMNG sample, synaptosomes were lysed in a modified buffer (20 mM Tris-HCl pH 8.0, 150 mM NaCl, 1% LMNG (w/v), 0.1% CHS (w/v) 200 μM NBQX, cOmplete EDTA-free protease inhibitors) for 3 hours at 4°C. Eluted protein was reduced with 5 mM dithiothreitol (DTT) at 56°C for 30 minutes and alkylated with 10 mM chloroacetamide at room temperature for 30 minutes. Excess chloroacetamide was quenched with 5 mM DTT for 10 minutes. 250ng of trypsin (Promega) was added and incubated at 37°C. After 4 hours, GDN was diluted to 0.003% and another 100 ng of trypsin was added for overnight digestion at 37°C. Digestion was stopped by the addition of formic acid (FA) to a final concentration of 0.5% and centrifuged at 18000 × g for 10 minutes to remove any particulate matter. Supernatants were desalted using home-made C18 stage tips (3M Empore), packed with 3 μL of Oligo R3 (Thermo Scientific) resin. Stage tips were equilibrated with 80% acetonitrile (MeCN)/0.5% FA followed by 0.5% FA. Bound peptides were eluted with 30-80% MeCN/0.5% FA and partially dried down in a vacuum concentrator (Savant).

All samples were analyzed by LC-MS/MS using a fully automated Ultimate 3000 RSLC nano System, fitted with a PepMap Neo C18 5 μm 0.3×5 mm nano trap column (Thermo Fisher Scientific) and an Aurora Ultimate TS 75μmx25cmx1.7μm C18 column (IonOpticks). Peptides were separated using buffer A (0.1% FA) and buffer B (80% MeCN, 0.1% FA) at flow rated of 300 nl/min and column temperature of 40°C. Eluted peptides were introduced directly via a nanoFlex ion source into a Q Exactive™ Plus Hybrid Quadrupole-Orbitrap™ Mass Spectrometer (Thermo Fisher Scientific). Mass spectrometer was set to a Data dependent acquisitions mode. MS1, full-scan (m/z 380-1600) with a resolution of 70K at 200 m/z, followed by MS2 acquisitions of the 15 most intense ions with a resolution of 35K and NCE (normalized collision energy) of 27%. MS1 target values of 1e6 and MS2 target values of 1e5 were used. The isolation window was set at 1.2 m/z and dynamic exclusion for 40 s.

The raw files were analyzed by MaxQuant (version 2.4.2.0) using standard settings and the LFQ and iBAQ options were selected. Spectra were submitted to database search against protein sequences from UP000008227_9823_sus scrofa (downloaded on July 2024) with the following parameters: up to two trypsin missed cleavage sites were allowed; carbamidomethylation of cysteine as fixed modification; and oxidation of methionine and acetylation N-terminal protein set as variable modifications. After checking for normal distribution, raw iBAQ values were then normalized to the mean value of the AMPAR core subunits (sum of GluA1-A4/4), as previously described (18, 95). Figures were prepared with GraphPad Prism v10.

### Single-cell RNA sequencing analysis

scRNA-seq data was extracted from a publicly available mouse cerebellum dataset (17), where raw data was normalized relative to each gene’s expression across all cell types in each annotation. Figures were made with GraphPad Prism v10.

### Molecular dynamic simulations

Molecular dynamics simulations of pig TARPs γ-7 and γ-2 were set up using initial models based on those from AlphaFold3 (96) as described in the cryo-EM data processing and model building section. The CHARMM-GUI Membrane Builder (97) was used to prepare the simulation systems. This included trimming the C-termini at residues 206 and 227 at the end of the helix for gamma-7 and gamma-2 respectively, neutralizing the truncated C-termini with N-methylamide caps (C-terminus patch CT3), orienting and positioning the protein in a bilayer with PPM building a 75 Å by 75 Å POPC bilayer around the protein using the pseudoatom replacement method (97) adding water and 0.15 M Na^+^ and Cl^-^ ions, performing an initial energy minimization in CHARMM and preparing simulation input files for GROMACS. To confirm that helix straightening was not an artefact of poor contacts, a second γ-7 system was prepared with interactions of Thr96 and Ser155 optimized in PyMOL 2.5 (Schrödinger, LLC) using mutagenesis and sculpting wizards, prior to CHARMM-GUI.

All molecular dynamics (MD) simulations were run using GROMACS 2021.5 (98) with the CHARMM36 all-atom force field (99), including the CHARMM36m update for proteins and the CHARMM TIP3P water model. Electrostatic interactions were treated with particle mesh Ewald using a short-range cut-off of 1.2 nm and van der Waals interactions used force switching between 1.0 and 1.2 nm, as recommended for CHARMM force fields in GROMACS. The simulation protocol in GROMACS was the standard one created by CHARMM-GUI. Energy minimization was run for 5000 steepest descent steps with position and dihedral restraints on the protein and lipid to relax the solvent around them. Harmonic position restraints were used for protein backbone and side-chain heavy atoms with force constants of 4000 and 2000 kJ·mol^−1^·nm^−2^ were in all directions, respectively. Lipids were treated with planar position restraints were used in the z-direction with force constants of 1000 kJ·mol^−1^·nm^−2^ to maintain lipid head groups in the planes of the membrane leaflets and harmonic dihedral restraints with force constants of 1000 kJ·mol^−1^·nm^−2^ to maintain correct orientation of phosphate groups and lipid tails relative to the core choline head group. These are close to the previously published values of 10.0, 5.0 and 2.5 kcal·mol^−1^·Å^−2^ (97). This was followed by several rounds of equilibration of different duration, gradually reducing the restraints as previously described (97). All steps after energy minimization used the Bussi-Donadio-Parrinello stochastic velocity rescaling thermostat with three coupling groups corresponding to protein, lipid and solvent atoms and a time constant of 0.1 ps and a target temperature of 303.15 K. The first two equilibration steps were in the NVT (constant number of atoms, volume and temperature) ensemble, only equilibrating the temperature. This was followed by four rounds of NPT (constant number of atoms, pressure and temperature) equilibration, adding semi-isotropic pressure equilibration with the Bernetti-Bussi stochastic cell rescaling barostat with a time constant of 5.0 ps and compressibility of 4.5 × 10^-5^ bar^-1^, equilibrating the system to a pressure of 1.0 bar, which was also maintained for all steps after NVT equilibration. Finally, the restraints were removed, and 100-200 ns production MD simulations were run in the NPT ensemble with the same thermostat and barostat with the same parameters. The first three equilibration steps used 1 fs time steps, after which 2 fs time steps where used. Bonds containing hydrogen were constrained with LINCS for all steps, including minimization.

## Supplementary Material

Movie S1

Movie S2

Movie S3

Movie S4

Supplementary Materials

Supp 1 data

## Data Availability

Cryo-EM coordinates and corresponding EM maps are deposited in the PDB and EMDB under the following accession codes - GluA1/4 NTD (EMDB-54547/PDB 9S3Q), GluA1/4 LBD (EMDB-54556/PDB 9S3Z), GluA1/4 TMD with γ7 (EMDB-54558/PDB 9S41) from the GluA2 depleted dataset; GluA2/4 NTD interface (EMDB-54543/PDB 9S3O); GluAx/A4 TMD with four TARPs (EMDB-54559) from the GluA2 un-depleted dataset recombinant GluA4 NTD (EMDB-55413) and recombinant GluA4-TARP γ2 LBD-TMD (EMDB-55414). Molecular dynamics runs are deposited on Zenodo with the following DOIs - TARP γ7 set 1 run 1 (10.5281/zenodo.17391587), TARP γ7 set 1 run 2 (10.5281/zenodo.17391645), TARP γ7 set 1 run 3 (10.5281/zenodo.17391694), TARP γ7 set 1 run 4 (10.5281/zenodo.17391745), TARP γ7 set 2 run 1 (10.5281/zenodo.17391300), TARP γ7 set 2 run 2 (10.5281/zenodo.17391401), TARP γ7 set 2 run 3 (10.5281/zenodo.17391530), TARP γ2 run 1 (10.5281/zenodo.17391807), TARP γ2 run 2 (10.5281/zenodo.17391826), TARP γ2 run 3 (10.5281/zenodo.17391843). The mass spectrometry proteomics data have been deposited to the ProteomeXchange Consortium via the PRIDE (100) partner repository with the dataset identifier PXD069620 and 10.6019/PXD069620. Source data are provided with this paper.

## References

[1] Hansen KB (2021). Structure, Function, and Pharmacology of Glutamate Receptor Ion Channels. Pharmacol Rev.

[2] Diering GH, Huganir RL (2018). The AMPA Receptor Code of Synaptic Plasticity. Neuron.

[3] Kessels HW, Malinow R (2009). Synaptic AMPA receptor plasticity and behavior. Neuron.

[4] Nicoll RA (2017). A Brief History of Long-Term Potentiation. Neuron.

[5] Park P (2018). The Role of Calcium-Permeable AMPARs in Long-Term Potentiation at Principal Neurons in the Rodent Hippocampus. Front Synaptic Neurosci.

[6] Greger IH, Watson JF, Cull-Candy SG (2017). Structural and Functional Architecture of AMPA-Type Glutamate Receptors and Their Auxiliary Proteins. Neuron.

[7] Jackson AC, Nicoll RA (2011). The expanding social network of ionotropic glutamate receptors: TARPs and other transmembrane auxiliary subunits. Neuron.

[8] Miguez-Cabello F (2025). GluA2-containing AMPA receptors form a continuum of Ca(2+)-permeable channels. Nature.

[9] Herguedas B (2019). Architecture of the heteromeric GluA1/2 AMPA receptor in complex with the auxiliary subunit TARP gamma8. Science.

[10] Zhao Y, Chen S, Swensen AC, Qian WJ, Gouaux E (2019). Architecture and subunit arrangement of native AMPA receptors elucidated by cryo-EM. Science.

[11] Yu J (2021). Hippocampal AMPA receptor assemblies and mechanism of allosteric inhibition. Nature.

[12] Zhang D, Watson JF, Matthews PM, Cais O, Greger IH (2021). Gating and modulation of a hetero-octameric AMPA glutamate receptor. Nature.

[13] Diaz-Alonso J (2017). Subunit-specific role for the amino-terminal domain of AMPA receptors in synaptic targeting. Proc Natl Acad Sci U S A.

[14] Stockwell I, Watson JF, Greger IH (2024). Tuning synaptic strength by regulation of AMPA glutamate receptor localization. Bioessays.

[15] Watson JF, Ho H, Greger IH (2017). Synaptic transmission and plasticity require AMPA receptor anchoring via its N-terminal domain. eLife.

[16] Keinanen K (1990). A family of AMPA-selective glutamate receptors. Science.

[17] Kozareva V (2021). A transcriptomic atlas of mouse cerebellar cortex comprehensively defines cell types. Nature.

[18] Schwenk J (2014). Regional diversity and developmental dynamics of the AMPA-receptor proteome in the mammalian brain. Neuron.

[19] Cathala L, Holderith NB, Nusser Z, DiGregorio DA, Cull-Candy SG (2005). Changes in synaptic structure underlie the developmental speeding of AMPA receptor-mediated EPSCs. Nat Neurosci.

[20] Kita K (2021). GluA4 facilitates cerebellar expansion coding and enables associative memory formation. Elife.

[21] Burnashev N (1992). Calcium-permeable AMPA-kainate receptors in fusiform cerebellar glial cells. Science.

[22] De Zeeuw CI, Hoogland TM (2015). Reappraisal of Bergmann glial cells as modulators of cerebellar circuit function. Front Cell Neurosci.

[23] Iino M (2001). Glia-synapse interaction through Ca2+-permeable AMPA receptors in Bergmann glia. Science.

[24] Saab AS (2012). Bergmann glial AMPA receptors are required for fine motor coordination. Science.

[25] Coombs ID, Cull-Candy SG (2009). Transmembrane AMPA receptor regulatory proteins and AMPA receptor function in the cerebellum. Neuroscience.

[26] Menuz K, O’Brien JL, Karmizadegan S, Bredt DS, Nicoll RA (2008). TARP redundancy is critical for maintaining AMPA receptor function. J Neurosci.

[27] Hashimoto K (1999). Impairment of AMPA receptor function in cerebellar granule cells of ataxic mutant mouse stargazer. J Neurosci.

[28] Noebels JL, Qiao X, Bronson RT, Spencer C, Davisson MT (1990). Stargazer: a new neurological mutant on chromosome 15 in the mouse with prolonged cortical seizures. Epilepsy Res.

[29] Sjostedt E (2020). An atlas of the protein-coding genes in the human, pig, and mouse brain. Science.

[30] Zhao Y, Chen S, Yoshioka C, Baconguis I, Gouaux E (2016). Architecture of fully occupied GluA2 AMPA receptor-TARP complex elucidated by cryo-EM. Nature.

[31] Tomita S (2003). Functional studies and distribution define a family of transmembrane AMPA receptor regulatory proteins. J Cell Biol.

[32] Yamazaki M (2010). TARPs gamma-2 and gamma-7 are essential for AMPA receptor expression in the cerebellum. Eur J Neurosci.

[33] Yamazaki M (2015). Relative contribution of TARPs gamma-2 and gamma-7 to cerebellar excitatory synaptic transmission and motor behavior. Proc Natl Acad Sci U S A.

[34] Dawe GB (2019). Nanoscale Mobility of the Apo State and TARP Stoichiometry Dictate the Gating Behavior of Alternatively Spliced AMPA Receptors. Neuron.

[35] Chen L (2000). Stargazin regulates synaptic targeting of AMPA receptors by two distinct mechanisms. Nature.

[36] Nakagawa T (2019). Structures of the AMPA receptor in complex with its auxiliary subunit cornichon. Science.

[37] Twomey EC, Yelshanskaya MV, Grassucci RA, Frank J, Sobolevsky AI (2017). Channel opening and gating mechanism in AMPA-subtype glutamate receptors. Nature.

[38] Vega-Gutierrez C (2025). GluA4 AMPA receptor gating mechanisms and modulation by auxiliary proteins. Nat Struct Mol Biol.

[39] Klykov O, Gangwar SP, Yelshanskaya MV, Yen L, Sobolevsky AI (2021). Structure and desensitization of AMPA receptor complexes with type II TARP gamma5 and GSG1L. Mol Cell.

[40] Douyard J, Shen L, Huganir RL, Rubio ME (2007). Differential neuronal and glial expression of GluR1 AMPA receptor subunit and the scaffolding proteins SAP97 and 4.1N during rat cerebellar development. J Comp Neurol.

[41] Gardinier KM (2016). Discovery of the First alpha-Amino-3-hydroxy-5-methyl-4-isoxazolepropionic Acid (AMPA) Receptor Antagonist Dependent upon Transmembrane AMPA Receptor Regulatory Protein (TARP) gamma-8. J Med Chem.

[42] Zhang D (2023). Modulatory mechanisms of TARP gamma8-selective AMPA receptor therapeutics. Nat Commun.

[43] Pokharna A (2025). Architecture, dynamics and biogenesis of GluA3 AMPA glutamate receptors. Nature.

[44] Rossmann M (2011). Subunit-selective N-terminal domain associations organize the formation of AMPA receptor heteromers. EMBO J.

[45] Zhao H (2017). Preferential assembly of heteromeric kainate and AMPA receptor amino terminal domains. Elife.

[46] Herguedas B, Krieger J, Greger IH (2013). Receptor heteromeric assembly-how it works and why it matters: the case of ionotropic glutamate receptors. Prog Mol Biol Transl Sci.

[47] Sobolevsky AI, Rosconi MP, Gouaux E (2009). X-ray structure, symmetry and mechanism of an AMPA-subtype glutamate receptor. Nature.

[48] Ivica J (2024). Proton-triggered rearrangement of the AMPA receptor N-terminal domains impacts receptor kinetics and synaptic localization. Nat Struct Mol Biol.

[49] Zhang D (2023). Structural mobility tunes signalling of the GluA1 AMPA glutamate receptor. Nature.

[50] Brorson JR, Li D, Suzuki T (2004). Selective expression of heteromeric AMPA receptors driven by flip-flop differences. J Neurosci.

[51] Coleman SK (2006). Isoform-specific early trafficking of AMPA receptor flip and flop variants. J Neurosci.

[52] Greger IH, Akamine P, Khatri L, Ziff EB (2006). Developmentally regulated, combinatorial RNA processing modulates AMPA receptor biogenesis. Neuron.

[53] Lomeli H (1994). Control of kinetic properties of AMPA receptor channels by nuclear RNA editing. Science.

[54] Mosbacher J (1994). A molecular determinant for submillisecond desensitization in glutamate receptors. Science.

[55] Sommer B (1990). Flip and flop: a cell-specific functional switch in glutamate-operated channels of the CNS. Science.

[56] Kato AS, Siuda ER, Nisenbaum ES, Bredt DS (2008). AMPA receptor subunit-specific regulation by a distinct family of type II TARPs. Neuron.

[57] Kato AS (2007). New transmembrane AMPA receptor regulatory protein isoform, gamma-7, differentially regulates AMPA receptors. J Neurosci.

[58] Soto D (2009). Selective regulation of long-form calcium-permeable AMPA receptors by an atypical TARP, gamma-5. Nat Neurosci.

[59] Jumper J (2021). Highly accurate protein structure prediction with AlphaFold. Nature.

[60] Koh DS, Burnashev N, Jonas P (1995). Block of native Ca(2+)-permeable AMPA receptors in rat brain by intracellular polyamines generates double rectification. J Physiol.

[61] Maher MP (2016). Discovery and Characterization of AMPA Receptor Modulators Selective for TARP-gamma8. J Pharmacol Exp Ther.

[62] Dzubay JA, Jahr CE (1999). The concentration of synaptically released glutamate outside of the climbing fiber-Purkinje cell synaptic cleft. J Neurosci.

[63] Larsen AH, Perozzo AM, Biggin PC, Bowie D, Kastrup JS (2024). Recovery from desensitization in GluA2 AMPA receptors is affected by a single mutation in the N-terminal domain interface. J Biol Chem.

[64] Watson JF, Pinggera A, Ho H, Greger IH (2021). AMPA receptor anchoring at CA1 synapses is determined by N-terminal domain and TARP gamma8 interactions. Nat Commun.

[65] Biederer T, Kaeser PS, Blanpied TA (2017). Transcellular Nanoalignment of Synaptic Function. Neuron.

[66] Nowacka A, Getz AM, Bessa-Neto D, Choquet D (2024). Activity-dependent diffusion trapping of AMPA receptors as a key step for expression of early LTP. Philos Trans R Soc Lond B Biol Sci.

[67] Hayashi Y (2000). Driving AMPA receptors into synapses by LTP and CaMKII: requirement for GluR1 and PDZ domain interaction. Science.

[68] Boudkkazi S (2023). A Noelin-organized extracellular network of proteins required for constitutive and context-dependent anchoring of AMPA-receptors. Neuron.

[69] Fang C (2025). Gating and noelin clustering of native Ca(2+)-permeable AMPA receptors. Nature.

[70] Delvendahl I, Hallermann S (2016). The Cerebellar Mossy Fiber Synapse as a Model for High-Frequency Transmission in the Mammalian CNS. Trends Neurosci.

[71] Silver RA, Traynelis SF, Cull-Candy SG (1992). Rapid-time-course miniature and evoked excitatory currents at cerebellar synapses in situ. Nature.

[72] Matsui K, Jahr CE, Rubio ME (2005). High-concentration rapid transients of glutamate mediate neural-glial communication via ectopic release. J Neurosci.

[73] Brusa R (1995). Early-onset epilepsy and postnatal lethality associated with an editing-deficient GluR-B allele in mice. Science.

[74] Zhu JJ, Esteban JA, Hayashi Y, Malinow R (2000). Postnatal synaptic potentiation: delivery of GluR4-containing AMPA receptors by spontaneous activity. Nat Neurosci.

[75] Pelkey KA (2015). Pentraxins coordinate excitatory synapse maturation and circuit integration of parvalbumin interneurons. Neuron.

[76] Aricescu AR, Lu W, Jones EY (2006). A time-and cost-efficient system for high-level protein production in mammalian cells. Acta Crystallogr D Biol Crystallogr.

[77] Elegheert J (2018). Lentiviral transduction of mammalian cells for fast, scalable and high-level production of soluble and membrane proteins. Nat Protoc.

[78] Scheres SH (2012). RELION: implementation of a Bayesian approach to cryo-EM structure determination. J Struct Biol.

[79] Zheng SQ (2017). MotionCor2: anisotropic correction of beam-induced motion for improved cryo-electron microscopy. Nat Methods.

[80] Punjani A, Rubinstein JL, Fleet DJ, Brubaker MA (2017). cryoSPARC: algorithms for rapid unsupervised cryo-EM structure determination. Nat Methods.

[81] Emsley P, Cowtan K (2004). Coot: model-building tools for molecular graphics. Acta Crystallogr D Biol Crystallogr.

[82] Yamashita K, Palmer CM, Burnley T, Murshudov GN (2021). Cryo-EM single-particle structure refinement and map calculation using Servalcat. Acta Crystallogr D Struct Biol.

[83] Croll TI (2018). ISOLDE: a physically realistic environment for model building into low-resolution electron-density maps. Acta Crystallogr D Struct Biol.

[84] Adams PD (2002). PHENIX: building new software for automated crystallographic structure determination. Acta Crystallogr D Biol Crystallogr.

[85] Pettersen EF (2004). UCSF Chimera--a visualization system for exploratory research and analysis. J Comput Chem.

[86] Davis IW, Murray LW, Richardson JS, Richardson DC (2004). MOLPROBITY: structure validation and all-atom contact analysis for nucleic acids and their complexes. Nucleic Acids Res.

[87] DeLano WL (2002).

[88] Dyer SC (2025). Ensembl 2025. Nucleic Acids Res.

[89] Edgar RC (2004). MUSCLE: multiple sequence alignment with high accuracy and high throughput. Nucleic Acids Res.

[90] Okonechnikov K, Golosova O, Fursov M, U team (2012). Unipro UGENE: a unified bioinformatics toolkit. Bioinformatics.

[91] Yariv B (2023). Using evolutionary data to make sense of macromolecules with a “face-lifted” ConSurf. Protein Sci.

[92] Garcia-Nafria J, Watson JF, Greger IH (2016). IVA cloning: A single-tube universal cloning system exploiting bacterial In Vivo Assembly. Sci Rep.

[93] Salazar H, Mischke S, Plested AJR (2020). Measurements of the Timescale and Conformational Space of AMPA Receptor Desensitization. Biophys J.

[94] Robert A, Howe JR (2003). How AMPA receptor desensitization depends on receptor occupancy. J Neurosci.

[95] Schwenk J (2012). High-resolution proteomics unravel architecture and molecular diversity of native AMPA receptor complexes. Neuron.

[96] Abramson J (2024). Accurate structure prediction of biomolecular interactions with AlphaFold 3. Nature.

[97] Jo S, Kim T, Im W (2007). Automated builder and database of protein/membrane complexes for molecular dynamics simulations. PLoS One.

[98] Pronk S (2013). GROMACS 4.5: a high-throughput and highly parallel open source molecular simulation toolkit. Bioinformatics.

[99] Vanommeslaeghe K, MacKerell AD (2015). CHARMM additive and polarizable force fields for biophysics and computer-aided drug design. Biochim Biophys Acta.

[100] Perez-Riverol Y (2025). The PRIDE database at 20 years: 2025 update. Nucleic Acids Res.

