# Supplementary material for: Structure and organization of AMPA receptor-TARP complexes in the mammalian cerebellum
Source: Science. Author manuscript; Available in PMC 2026 May 27. (PMC7619101; doi:10.1126/science.aeb3577)
Supplement: Supplementary Materials [file EMS213805-supplement-Supplementary_Materials.docx]

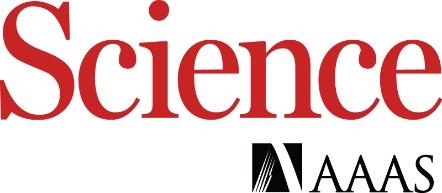


Supplementary Materials for

**Structure and organization of AMPA receptor-TARP complexes**

**in the mammalian cerebellum**

Alexander M. Scrutton^1,^†, Nayanika Sengupta^1,^†, Josip Ivica^1,^†, Imogen Stockwell^1^, Sew Peak-Chew^2^, Bishal Singh^3^, Kunimichi Suzuki^4^, Veronica T. Chang^1^, Stephen H. McLaughlin^5^, James M. Krieger^1^, A. Radu Aricescu^1^, Ingo H Greger^1,^*

Corresponding author: ig@mrc-lmb.cam.ac.uk

**The PDF file includes:**

Figs. S1 to S10

Tables S1 to S2

**Other Supplementary Materials for this manuscript include the following:**

Movies S1 to S4

Data S1


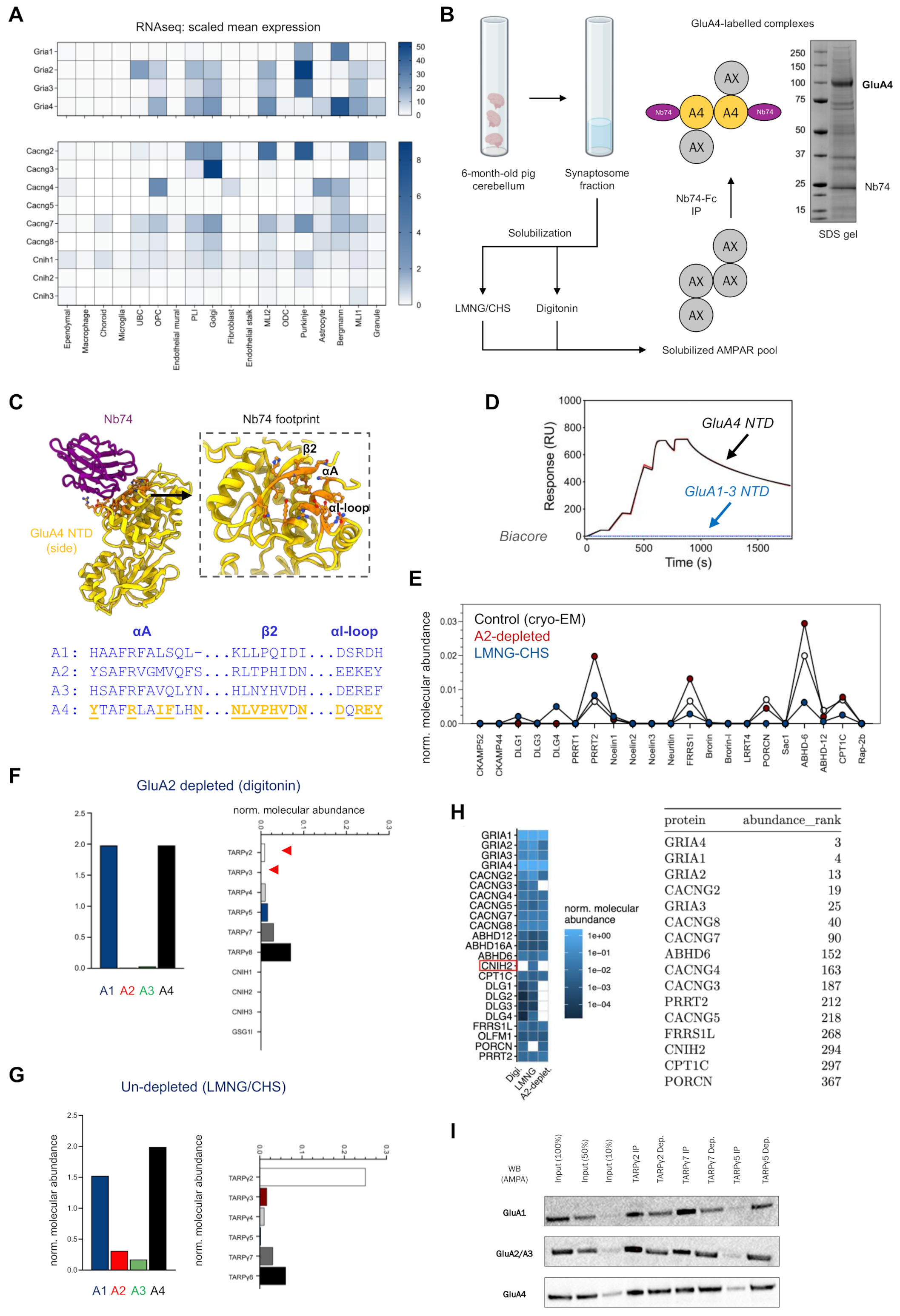


**Fig. S1. Transcriptomic and proteomics of cerebellar AMPARs.** (**A**) RNAseq analysis of AMPAR components from mouse cerebellum. Scaled mean expression values from (*17*) were used to generate the figure. TARP−γ2 and TARP− γ7 dominate in various cell types, as well as GluA4. (**B**) Overview of the purification procedure used to extract native GluA4 complexes from pig cerebellum. Synaptosome fractions were generated from cerebellum homogenates, which were then solubilized in a lysis buffer containing either digitonin (1% w/v) or LMNG/CHS (1/0.1% w/v). The solubilized AMPAR pool was then immunoprecipitated with the GluA4 specific nanobody. Representative denaturing SDS-PAGE gel shows a clear enrichment of AMPARs at the ∼100 kDa band. Synaptosome diagram created with BioRender.com (**C**) Crystal structure of Nb74 (purple) bound to the GluA4 NTD (yellow), the interaction footprint of the NTD is shown in orange. The GluA1-4 sequence alignment covers the footprint area, demonstrating GluA4 specificity. (**D**) Surface plasmon resonance (SPR) analysis of the Nb74 interaction with GluA4-NTD. Biotinylated GluA4-NTD was immobilized onto streptavidin-coated chips. In the first single-cycle kinetic (SCK1) experiment, a 1:3 dilution series of 600 nM Nb74, was injected in increasing concentration order. The highest concentration was subsequently reduced for SCK2 and SCK3 to 100 nM. Data were double referenced from signals on a reference channel immobilized with GluA2-NTD and a buffer blank. The kinetic data from the three experiments were averaged to calculate a Kd of 0.60 ± 0.13 nM. (**E**) Semi-quantitative mass spectrometry data of purified GluA4-complexes from pig cerebellum showing distribution of auxiliary AMPAR subunits across purification conditions. (**F**) Semi-quantitative mass spectrometry data of digitonin solubilized and GluA2 depleted cerebellar GluA4-complexes, showing distribution of core and auxiliary AMPAR subunits from GluA4 immunoprecipitation after GluA2 depletion. Red triangles highlight decrease in amount of TARPs γ2 and γ3 upon GluA2 depletion. (**G**) Semi-quantitative mass spectrometry data of LMNG/CHS solubilized cerebellar GluA4-complexes, showing distribution of core and auxiliary AMPAR subunits after GluA2 immunoprecipitation. (**H**) Summary of MS data from GluA4 pull-down across conditions, highlighted small CNIH abundance in LMNG solubilized samples. White boxes denote ‘undetected’ signal. (**I**) TARP IP depletion experiments highlighted the co-localization of type I TARPs (γ2) with GluA2 and GluA3, while type II TARPs (γ7) segregate with GluA4 and GluA1.


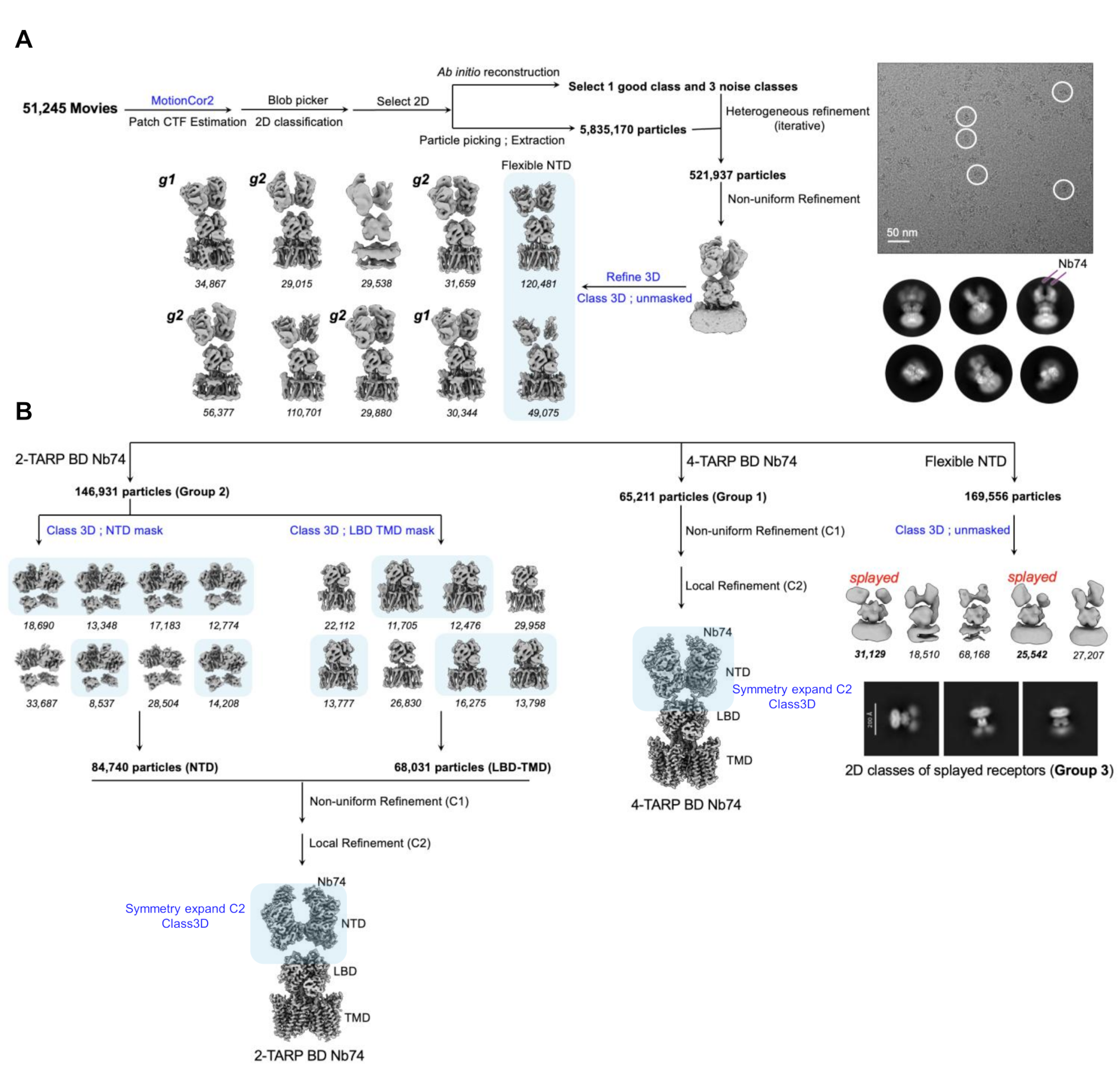


**Fig. S2. Cryo-EM data processing pipeline for cerebellar GluA4 proteome.** (**A**) Overview of the cryo-EM data processing. Representative raw micrograph and 2D class averages showing diverse particle orientations of the receptor. Density corresponding to GluA4 nanobody Nb74 has been marked with purple lines. (**B**) Different groups of cerebellar receptors comprise AMPARs with 4-TARPs (group 1) or 2-TARPs (group 2). Majority of the receptors have a compact well-resolved NTD (groups 1 and 2), while a small proportion of receptors show flexible and splayed NTDs (group 3), indicating further AMPAR diversity. Blue indicates jobs run in RELION and black denotes jobs run in CryoSPARC. See methods for processing details.


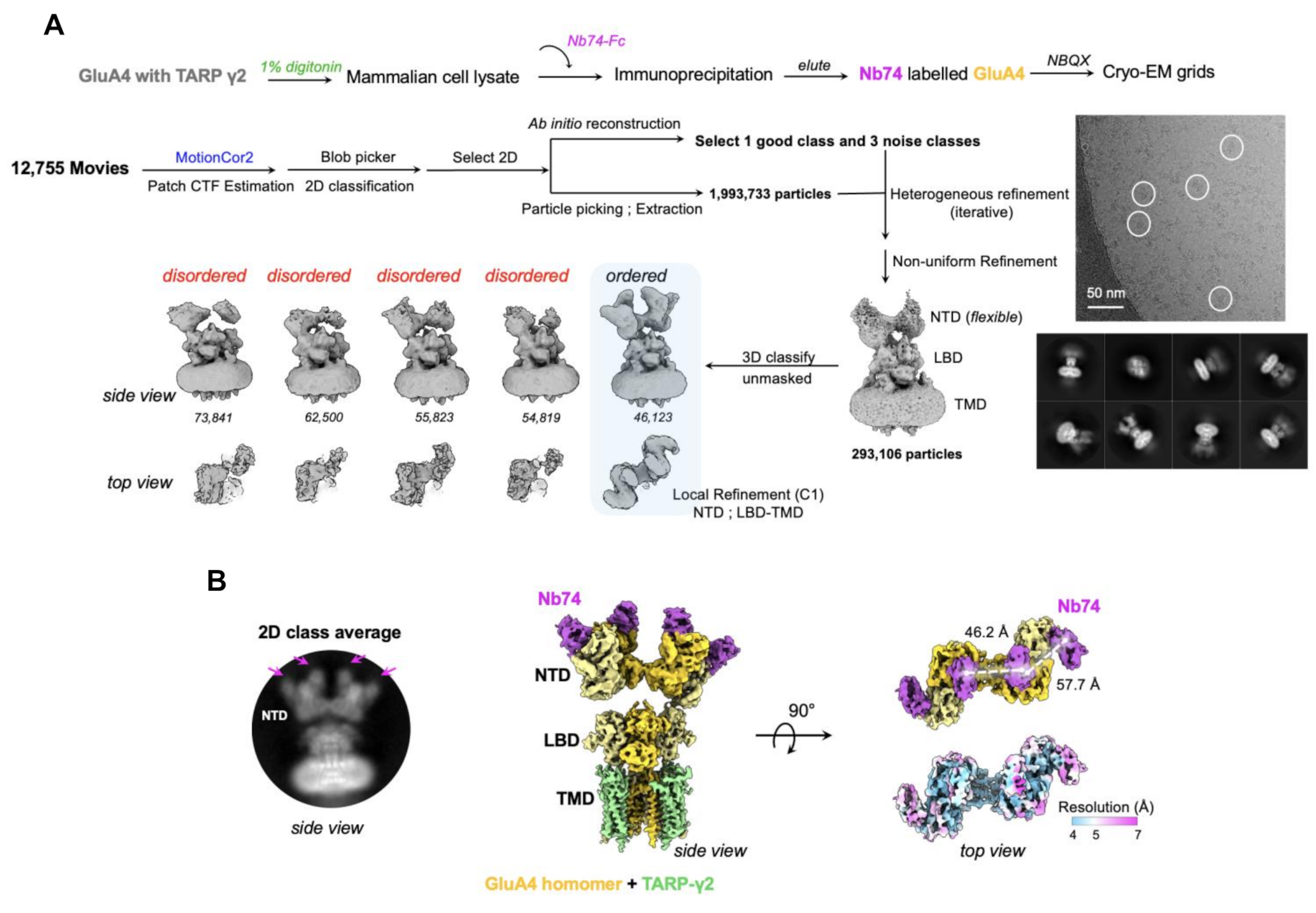


**Fig. S3. Cryo-EM data processing pipeline for recombinant GluA4 purified with Nb74.** (**A**) Schematic representation showing the protocol used to isolate recombinantly expressed GluA4 homomers with the help of nanobody Nb74. Majority of the receptors exhibit dynamic NTD as observed in the 2D and 3D classes. (**B**) Cryo-EM structure of recombinant GluA4 homomer. Left: 2D class average of a side of the GluA4 homomeric receptor. Pink arrows show densities for Nb74 on all four NTDs. Right: A non-symmetrized composite map of GluA4 (yellow) in complex with TARP-γ2 (green). Local resolution map of the non-symmetrized locally refined N-terminal domain shows that the resolution of the Nb74 is comparable with each other and the receptor.


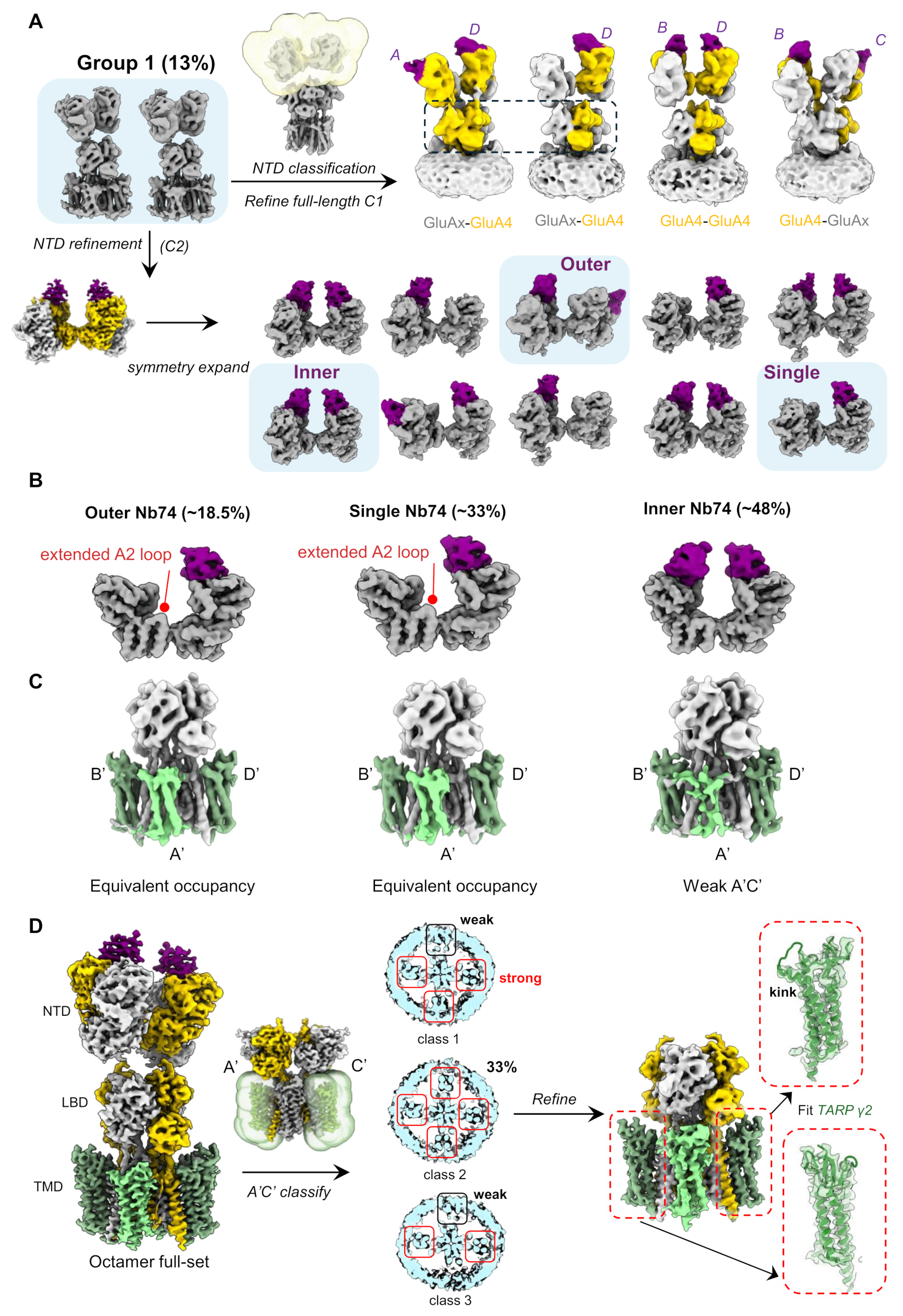


**Fig. S4. Compositional heterogeneity within the Group 1 receptors.** (**A**) The fully TARP-ed group 1 receptors representing 13% of the total isolated AMPARs were initially classified focused on their NTD. Asymmetric full-length reconstructions of the obtained classes represent different sites of GluA4 occupancy (coloured yellow in the maps). Black dashed box has been used to compare a GluA4 homomeric LBD vs a GluA4/AX heteromeric LBD. NTD of the entire group 1 receptors was refined to high-resolution (with C2 symmetry imposed), followed by symmetry expansion and NTD focused classification to identify classes with heterogeneous nanobody binding. (**B**) Three types of receptors obtained after symmetry expansion, where majority of the receptors have two GluA4 subunits at the B/D positions. Receptors lacking a GluA4 subunit at one of the B/D sites show an extended loop (marked with red line) after helix F. (**C**) TARP (coloured green) sectors for each of the receptor types shows correlation of TARP strength with nanobody/GluA4 occupancy. (**D**) A subset of the group 1 receptors, containing equally strong TARPs at all four auxiliary binding sites was refined to identify the TARP type. TARP-γ2 from PDB 9B5Z*(48)* was used to fit into B’D’ TARP density.


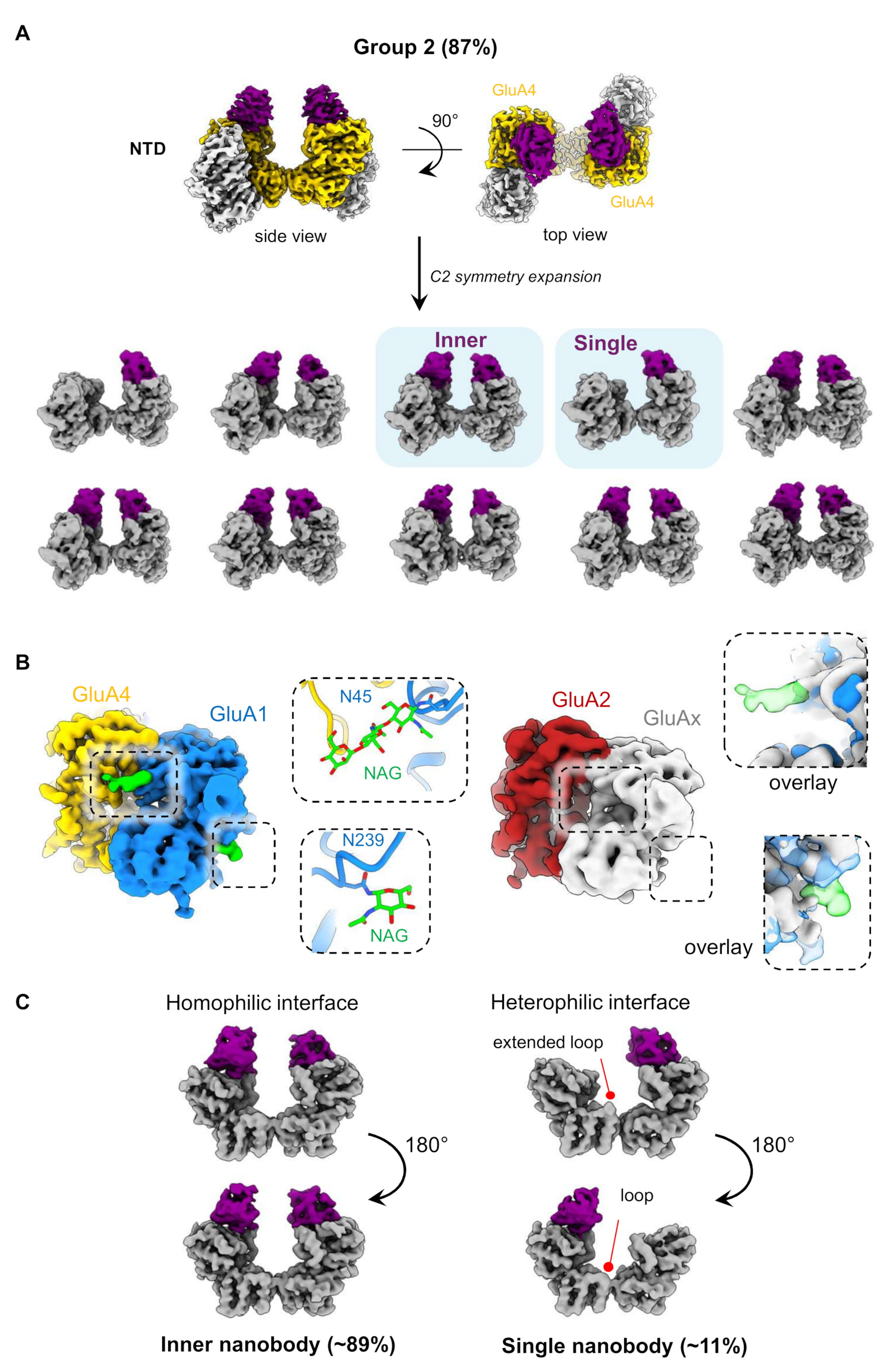


**Fig. S5. Composition of the 2-TARPed AMPARs.** (**A**) High-resolution NTD reconstruction of the group 2 receptors (representing 87% of the total isolated AMPARs) was symmetry expanded and classified. NTDs containing either two or one central nanobody, were obtained. (**B**) Receptor containing two central nanobodies was identified to be a GluA1/A4 complex (left) based on the presence of two GluA1-specific N-linked glycans at N45 and N239, respectively. Receptor containing a single nanobody (right) was identified as a GluAX-A2-A4-AX type. Lack of observable glycan density prevented identification of the A/C subunits. (**C**) Comparison between the A4-A4 homophilic interface and A2-A4 heterophilic interface shows an extended loop (marked with red line) after helix F, a feature characteristic of GluA2.

**
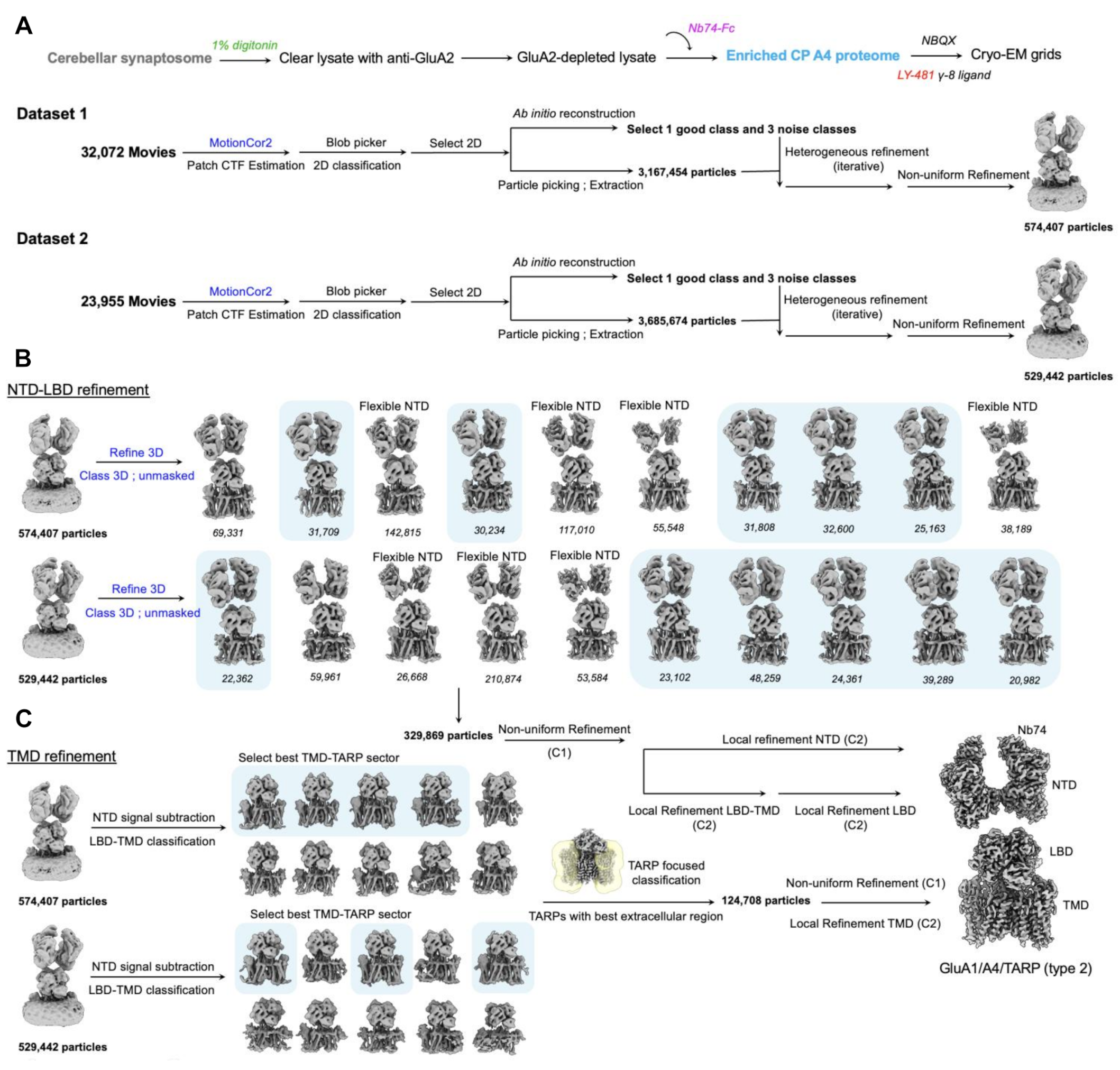
**

**Fig. S6. Cryo-EM data processing pipeline for cerebellar GluA4 proteome after GluA2 depletion.** (**A**) Schematic showing the purification procedure followed to obtain GluA4-enriched calcium-permeable AMPAR pool. Two datasets from independent purifications were processed. (**B**) All classes show the presence of only 2-TARPed receptors. To produce high-resolution NTD and LBD maps, classes showing the best features (nanobody and A1-specific glycan) were selected. (**C**) For the TMD sector, classes showing the best-resolved TARP extracellular domains were pooled and refined. Blue indicates jobs run in RELION and black denotes jobs run in CryoSPARC. See methods for processing details.


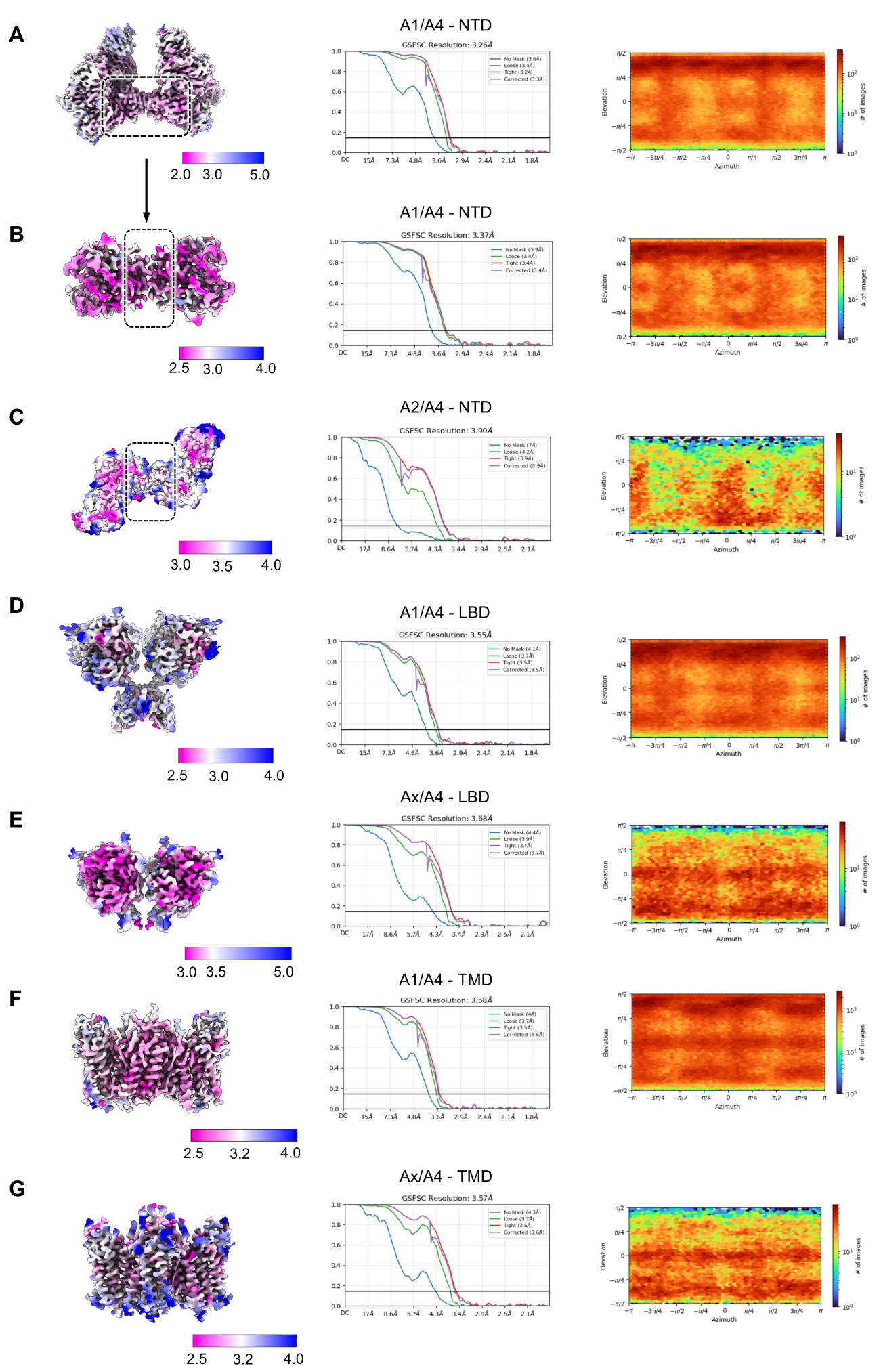


**Fig. S7. Local resolution and overall map quality of the GluA1/A4 receptor and GluA2/4 NTD interface.** (**A-G**), Local resolution maps computed for each voxel, where resolutions were computed at Fourier shell correlation (FSC) = 0.143. Gold standard Fourier shell correlation (GSFSC) curves for each map are shown on the middle lane of each panel. Black line is FSC = 0.143. Y axis is FSC, X is resolution in Å. Heat maps of particle orientation distribution for each structure are shown on the right lane of each panel.


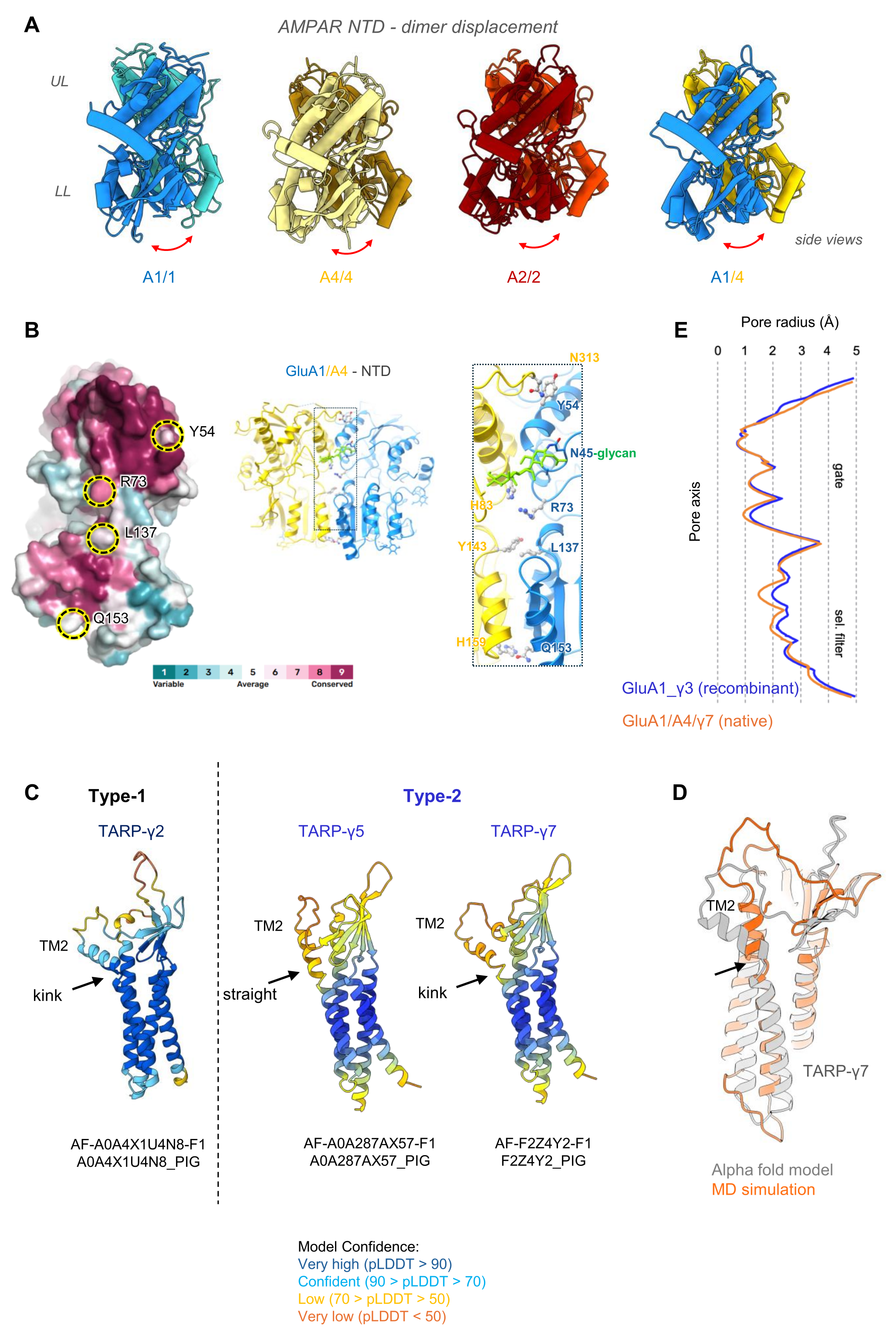


**Fig. S8. Comparative analysis of AMPAR NTD dimers and architecture of Type 1 and Type 2 TARPs.** (**A**) Side view of different AMPAR NTD dimers show displacement of one of the dimers with respect to the other. The PDBs used for A1/1, A4/4 and A2/2 are 3SAJ , 4GPA and 9B68, respectively. (**B**) Conservation of the NTD dimer interface generated by Consurf (*91*), using 165 mammalian GluA1-4 sequences. The greater sequence conservation in the upper part of the interface is highlighted (conservation score '9'; maroon). This conserved pact is interrupted by Y54 and H183 (circled). The lower part is less well conserved (see scale bar on the right). (**C**) AlphaFold models of TARPs γ2, γ5, and γ7 (left), highlighting a ‘kink’ in the transmembrane helix 2 of TARP γ2 and potentially in TARP γ7. Structures were modelled in AlphaFold 3 using Sus scrofa UniProt sequences (IDs mentioned in the figure). Confidence of model is shown from red (low) to blue (high). (D). A snapshot from molecular dynamics simulations of TARP γ7 (right, orange) aligned to the AlphaFold starting model (grey) shows straightening of the TARP γ7 TM2 helix. (E) Comparison of the pore profiles of recombinant GluA1_TARP-γ3 in a resting state (blue) and native GluA1/A4/TARP-γ7 (orange).

**
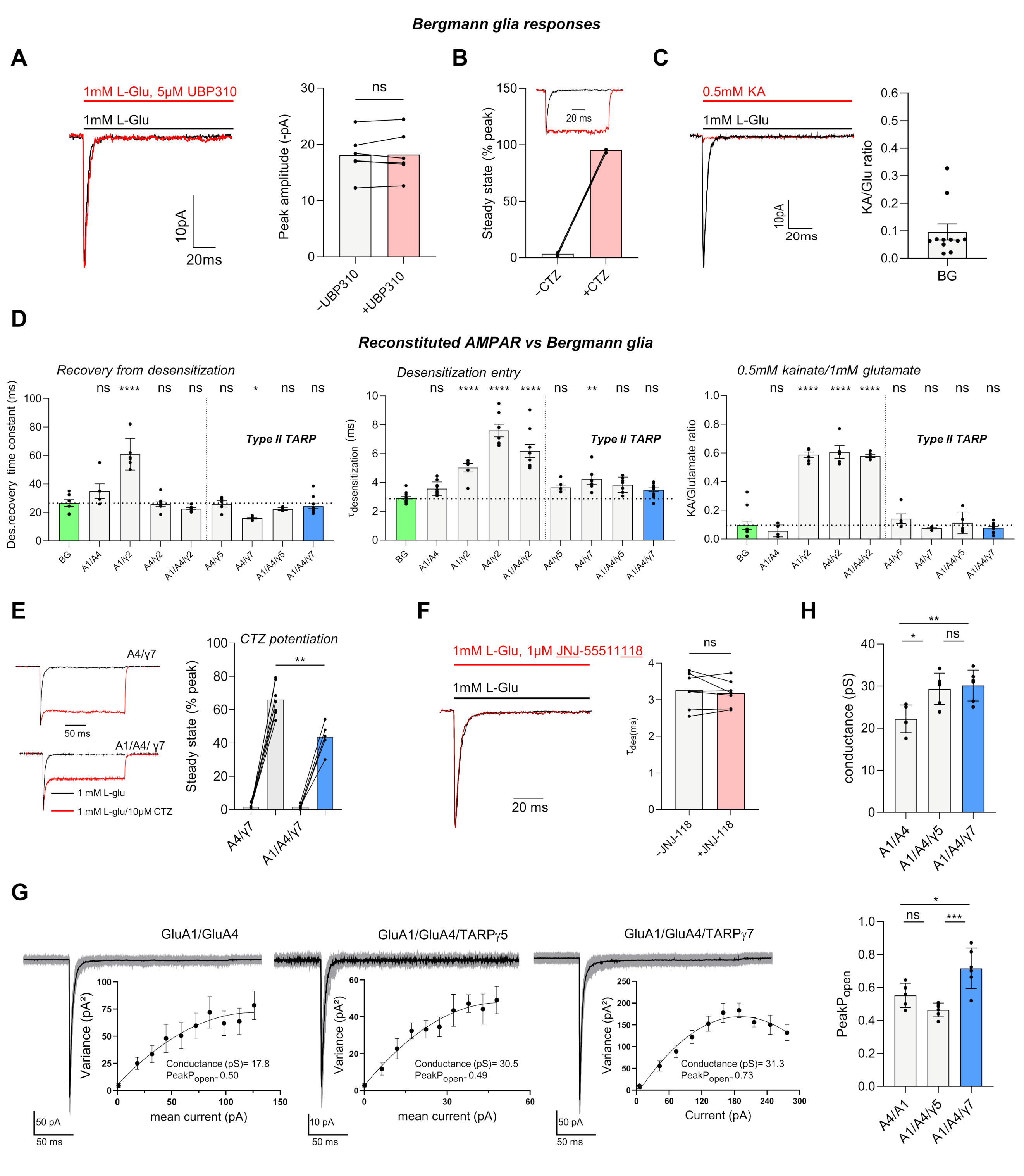
**

**Fig. S9. Characterization of Bergmann glia responses.** (**A**) Current trace from an outside-out patch from a Bergmann glial soma showing response to 1 mM glutamate (black), unaffected by 5 µM UPB310 (red). Right, peak currents evoked by glutamate alone (grey) or with UPB310 (red); n = 6 patches, paired recordings. (**B**) Bar plot showing steady-state potentiation of BG AMPAR currents by 100 µM cyclothiazide (CTZ). Inset: representative traces in response to glutamate alone (black) or with CTZ (red). (**C**) Current BG patch evoked by 1 mM glutamate or 0.5 mM kainate. Bar graph shows the distribution of relative peak amplitudes. (**D**) Kinetic parameters from outside-out patches of Bergmann glia (BG, yellow) and HEK293T cells expressing AMPAR/TARP combinations. Left, recovery from desensitization: BG (n = 7), GluA1/GluA4 (n = 5), GluA1/γ2 (n = 6), GluA4/γ2 (n = 9), GluA1/GluA4/γ2 (n = 6), GluA4/γ5 (n = 5), GluA4/γ7 (n = 6), GluA1/GluA4/γ5 (n = 7), GluA1/GluA4/γ7 (n = 11). One-way ANOVA, F_(8,53)_=26.85, P<0.0001; Dunnett’s test: ****P<0.0001, P = 0.0206, ns = not significant. Middle, entry into desensitization for BG (n = 11) and recombinant receptors (n = 5–11); F_(8,64)_=31.77, P<0.0001; ****P<0.0001, **P= 0.0082. Right, kainate/glutamate peak current ratios for BG (n = 11) and recombinant receptors (n = 5–11); F_(8,49)_=86.54, P<0.0001; ****P<0.0001. (**E**) Currents from outside-out patches of HEK293T cells expressing GluA4/γ7 or GluA1/GluA4/γ7 in response to 1 mM glutamate (black) or with 10 μM CTZ (red). Right, steady-state potentiation was greater in GluA4/γ7 (n = 7) than GluA1/GluA4/γ7 (n = 6); t = 4.433, df = 11, P = 0.0012. (**F**) Current from BG patch evoked with 1 mM glutamate alone (black) or coapplied with 1 µM JNJ-118(red). Right, desensitization time constants of control vs. 1 µM JNJ-118 (n = 7); no significant difference (paired t-test). (**G)** Representative currents and NSFA plots from HEK293T patches expressing GluA1/GluA4, GluA1/GluA4/γ5, or GluA1/GluA4/γ7 in response to 10 mM glutamate (200 ms, -60 mV). Insets: current–variance plots showing single-channel conductance (γ) and peak open probability (Po). (**H**) Summary of NSFA parameters: weighted conductance (top) and Po (bottom). GluA1/GluA4 (n = 5), GluA1/GluA4/γ5 (n = 5), GluA1/GluA4/γ7 (n = 6). Weighted conductance; One-way ANOVA, F_(2, 14)_ = 7.797, P=0.0053; Tukey’s test: **P = 0.0071, *P = 0.0145, ns = not significant. PeakP_open_: One-way ANOVA, F_(2,14)_ =12.88, P=0.0007; Tukey’s test: ***P = 0.0005, *P =0.0198.


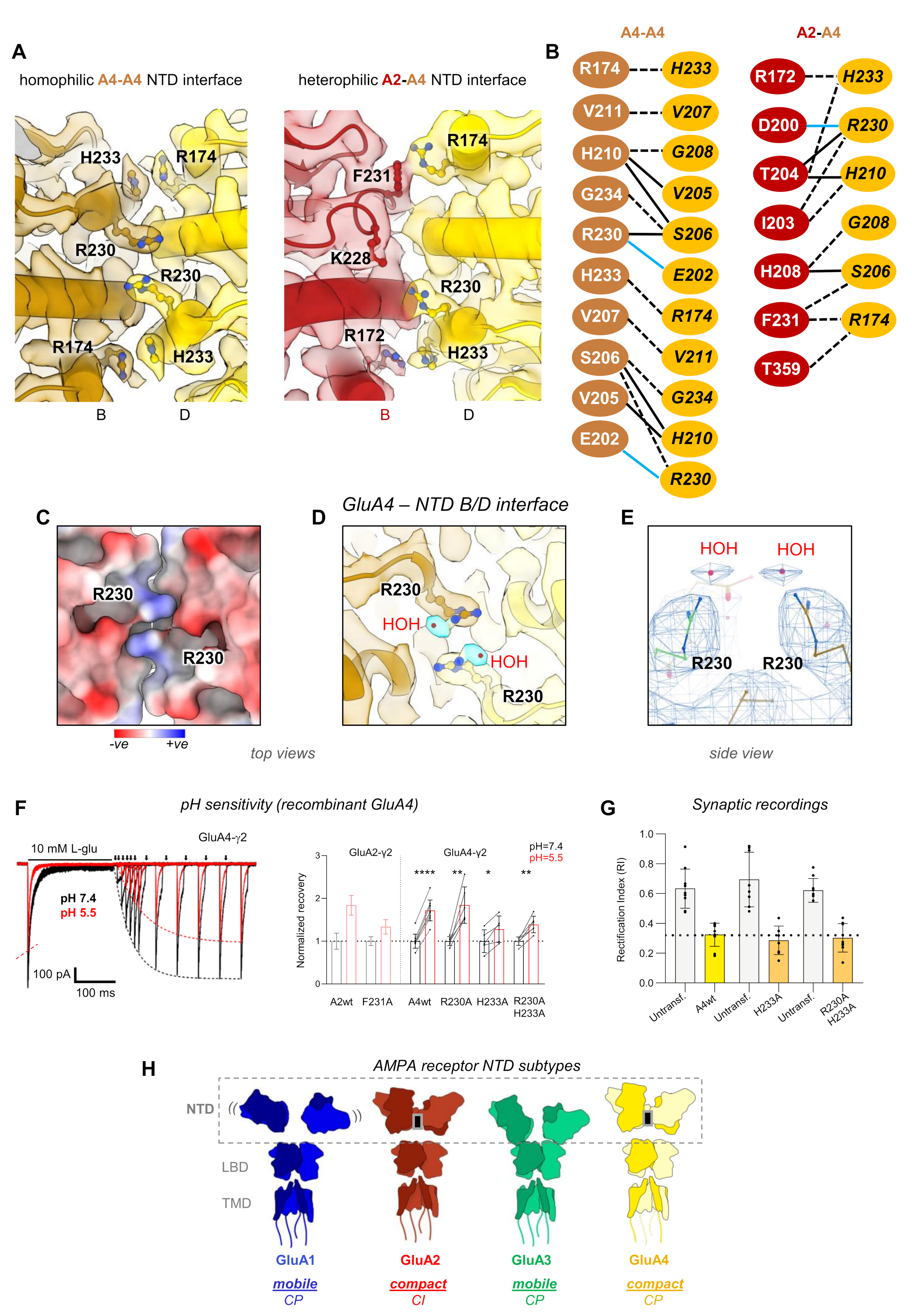


**Fig. S10.** **Features of the CP and CI AMPAR NTD tetrameric interface.** (**A**) Cryo-EM map of the GluA4/A4 and GluA2/A4NTD interface, with key interface-forming residues highlighted. (**B**) PDB sum interface map showing contacting residues in both interfaces, highlighting the extensive interaction network in GluA4/A4 compared to GluA4/A2. Dashed lines represent non-bonded contacts, black solid lines represent hydrogen bonds and blue solid lines denote salt-bridge interactions. (**C**) Electrostatic potential surface of the homophilic GluA4/A4 interface shows the central R230 from each GluA4 subunit. (**D, E**) Water molecules coordinate the R230 bridge in the GluA4/A4 interface, as observed in refined cryo-EM structure. (**F**) Recovery from desensitization of GluA4/TARPy2 at pH 7.4 (black trace) and 5.5 (red traces). Dashed lines represents the Hodgkin-Huxley fit, τ_rec(pH7.4)_=24ms, τ_rec(pH5.5)_=42ms. Recovery from desensitization (normalized to pH 7.4) measured from outside-out patches expressing for GluA4-γ2 constructs at pH 7.4 (black) and pH 5.5 (red). n = 11 (WT), 6 (R230A), 5 (H233A), 6 (R230A/H233A). Paired t-tests: **P<0.001, *P=0.028, P=0.0124, *P=0.0016 (left to right). Data for GluA2 are from (*48*).(**G**) Synaptic incorporation of transfected AMPARs in organotypic slices, indicated by a reduced rectification index relative to a simultaneously recorded adjacent untransfected neuron. The line represents the mean rectification index from neurons transfected with A4_wt_. (**H**) NTD-based classification of AMPAR subunits. Compact NTDs characterize CI GluA2 and CP GluA4 (yellow); splayed NTDs are typical of CP GluA1 and GluA3.

|  | GluA1/4 NTD tetramer  (EMDB-54547)  (PDB 9S3Q) | GluA1/4 LBD tetramer  (EMDB-54556)  (PDB 9S3Z) | GluA1/4 TARP γ7 TMD  (EMDB-54558)  (PDB 9S41) | GluA2/4 NTD interface  (EMDB-54543)  (PDB 9S3O) | GluAx/4 TARP TMD  (EMDB-54559) | Recombinant GluA4 NTD  (EMD-55413) | Recombinant GluA4 LBD-TMD + TARP γ2  (EMD-55414) |
| --- | --- | --- | --- | --- | --- | --- | --- |
| **Data collection and processing** |  |  |  |  |  |  |  |
| Microscope | TFS Titan Krios | TFS Titan Krios | TFS Titan Krios | TFS Titan Krios | TFS Titan Krios | TFS Titan Krios | TFS Titan Krios |
| Detector | BioQuantum K3+GIF | BioQuantum K3+GIF | BioQuantum K3+GIF | Falcon4i Selectris X | Falcon4i  Selectris X | Falcon4i  Selectris X | Falcon4i  Selectris X |
| Magnification | 105,000x | 105,000x | 105,000x | 130,000x | 130,000x | 130,000x | 130,000x |
| Voltage (kV) | 300 | 300 | 300 | 300 | 300 | 300 | 300 |
| Electron exposure (e^–^/Å^2^) | 40 | 40 | 40 | 40 | 40 | 40 | 40 |
| Defocus range (μm) | -1.2 to -2.4 | -1.2 to -2.4 | -1.2 to -2.4 | -1.2 to -2.4 | -1.2 to -2.4 | -1.2 to -2.4 | -1.2 to -2.4 |
| Pixel size (Å) | 0.826 | 0.826 | 0.826 | 0.955 | 0.955 | 0.955 | 0.955 |
| Symmetry imposed | C2 | C2 | C2 | C1 | C2 | C1 | C1 |
| Initial particle images (no.) | 1,103,849 | 1,103,849 | 1,103,849 | 521,937 | 521,937 | 293,106 | 293,106 |
| Final particle images (no.) | 329,869 | 329,869 | 124,708 | 30,603 | 65,211 | 46,065 | 46,065 |
| Map resolution (Å)  FSC threshold | 3.26  0.143 | 3.55  0.143 | 3.66  0.143 | 3.90  0.143 | 3.57  0.143 | 4.23  0.143 | 3.64  0.143 |
| Map resolution range (Å) | 2.5-6.5 | 2.8-7 | 2.7-8.2 | 3.3-5.5 | 3-8.5 | 4-7.5 | 3-7.2 |
| **Refinement** |  |  |  |  |  |  |  |
| Initial model used (PDB code) | AF-I3L8N9-F1, AF-A0A286ZS63-F1 | AF-I3L8N9-F1, 7OCE | AF-P62956-F1, 7OCE | AF-I3L8N9-F1, 9B68 | - | - | - |
| Model resolution (Å)  FSC threshold | 3.3  0.143 | 3.5  0.143 | 3.6  0.143 | 4.2  0.143 | -  0.143 | -  0.143 | -  0.143 |
| Map sharpening *B* factor (Å^2^) | -132.4 | -138.2 | -161.3 | -110 | -107.6 | -130.1 | -76.7 |
| Model composition  Non-hydrogen atoms  Protein residues  Ligands | 11632  1454  NAG:16  BMA:2 | 8263  1087  0 | 6891  916  - | 5384  700  NAG:1 | -  -  - | -  -  - | -  -  - |
| *B* factors (Å^2^)  Protein (mean)  Ligand (mean) | 51.52  87.19 | 64.46  - | 106.40  - | 56.84  53.09 | -  - | -  - | -  - |
| R.m.s. deviations  Bond lengths (Å)  Bond angles (°) | 0.003  0.541 | 0.003  0.638 | 0.002  0.464 | 0.003  0.642 | -  - | -  - | -  - |
| Validation  MolProbity score  Clashscore  Poor rotamers (%) | 1.02  2.37  0.00 | 1.39  7.15  0.48 | 1.19  4.04  0.29 | 1.58  6.68  0.00 | -  -  - | -  -  - | -  -  - |
| Ramachandran plot  Favored (%)  Allowed (%)  Disallowed (%) | 98.32  1.68  0 | 98.13  1.87  0 | 99.43  0.57  0 | 96.66  3.34  0 | -  -  - | -  -  - | -  -  - |
|  |  |  |  |  |  |  |  |

**Table S1. Cryo** **EM data processing and refinement statistics.**

|  | _τw, desensitization (ms)_  _(n)_ | _τ recovery (ms)_  _(n)_ | _Steady-state_  _(n)_ | _Peak 1/10 mM L-glut_  _(n)_ | _Deactivation (ms)_  _(n)_ | _Rise time (ms)_  _(n)_ |
| --- | --- | --- | --- | --- | --- | --- |
| Bergmann glia | 2.9± 0.30  (11) | 27.0 ±6.3  (7) | 1.0 ± 0.35  (7) | 0.35 ± 0.12  (7) | 0.69 ± 0.18  (6) | 0.21 ± 0.07  (7) |
| Recombinant AMPARs (A1flop;A4 flip) | | | | | | |
| A1/A4 | 3.6±0.47  (10) | 34.9±11.7  (5) | 0.50± 0.3  (10) | 0.38± 0.05  (5) | 0.37± 0.06  (9) | 0.16 ± 0.02  (5) |
| A1/γ2 | 5.0±0.74  (6) | 60.9±11.1  (6) | 1.0± 0.8  (6) | 0.76 ± 0.04  (6) | 1.2 ± 0.03  (6) | 0.10 ± 0.01  (6) |
| A4/γ2 | 7.5±1.2  (7) | 24.5±6.6  (5) | 3.6± 1.1  (7) | 0.70± 0.03  (4) | 0.89 ± 0.16  (9) | 0.11 ± 0.03  (7) |
| A1/A4/γ2 | 5.8 ±1.4  (8) | 22.6±2.1  (6) | 1.9 ± 0.9  (8) | 0.70± 0.02  (4) | 0.97 ± 0.22  (6) | 0.10 ± 0.02  (6) |
| A1/γ5## | 2.3±0.3  (5) | 68.0± 3.0  (5) | 0.28± 0.18  (5) | 0.13 ± 0.02  (5) | nd | nd |
| A1/γ7## | 3.5±0.14  (5) | 63.2±3.8  (5) | 1.1± 0.9  (5) | 0.62 ± 0.05  (5) | nd | nd |
| A4/γ5 | 3.7±0.4  (6) | 26.0±4.9  (5) | 0.8± 0.5  (6) | 0.14± 0.02  (5) | 0.58± 0.16  (5) | 0.26 ± 0.05  (6) |
| A4/γ7 | 4.2±0.9  (6) | 15.8.±1.4  (6) | 1.2± 0.8  (6) | 0.27± 0.03  (5) | 0.66± 0.07  (5) | 0.12 ± 0.01  (6) |
| A1/A4/γ5 | 3.8±0.53  (7) | 22.4±1.1  (7) | 0.59± 0.6  (7) | 0.17± 0.05  (5) | 0.67± 0.20  (5) | 0.24 ± 0.03  (7) |
| A1/A4/γ7 | 3.5±0.47  (11) | 24.4±5.7  (11) | 0.5± 0.4  (11) | 0.34± 0.07  (11) | 0.77± 0.17  (11) | 0.14 ± 0.04  (11) |

**Table S2. Outside-out current parameters measured for native BG and recombinant AMPARs.** Values are mean± s.d. ##Values were obtained from the whole cell recordings.

**Supplementary movie legends**

**Movie S1.**

Snapshots from a 200-ns molecular dynamics simulation of TARP-γ7 in a POPC membrane (not shown), highlighting formation and stabilization of an upright transmembrane helix 2 in blue.

**Movie S2.**

Snapshots from the first 50 ns of a molecular dynamics simulation of TARP-γ2 in a POPC membrane (not shown), highlighting stability of a kinked transmembrane helix 2 in blue.

**Movie S3.**

A rotating map of the Type I octameric CI GluA2/A4 receptor typically found in granule cells, giving a better view of the main interfaces. The cryo-EM map is coloured as in Fig.1D, I with unidentified chains A and C in grey and GluA2 chain B in red and GluA4 chain D in yellow with the nanobody in purple and the TARPs in 2 shades of green. Cyan has been used to colour the GluA2-specific loop extending after helix F. It is rotated between different views in Figure 1 to give a better impression of its structure in three dimensions and the relationship between the different interfaces.

**Movie S4.**

A rotating map of the Type 2 hexameric CP GluA1/A4 receptor characteristic of Bergmann Glia, giving a better view of the main interfaces. The cryo-EM map is coloured as in Fig. 2 with GluA1 chains A+C in blue and GluA4 chains B+D in yellow with the nanobody in purple and the 2 TARPs in pink. It is rotated between different views in Figure 2 to highlight the N-terminal domain (NTD) interface and the extended extracellular region (ECR) characteristic of Type 2 TARPs like TARP- γ7.

**Data S1.**

Raw electrophysiological data and statistics.
